# Synthesis of magnetic borosilicate zeolite/graphene quantum dots nanocomposites for removal of nitrate and organic pollutants from water

**DOI:** 10.1038/s41598-025-07746-4

**Published:** 2025-07-01

**Authors:** Robab Shahi, Maasoumeh Khatamian

**Affiliations:** Department of Inorganic Chemistry, Faculty of Chemistry, University of Tabriz, Tabriz, Iran

**Keywords:** Borosilicate zeolite, ZSM-5, Graphene quantum Dots, Magnetic nanocomposite, Adsorbent, Environment remediation, Fe_3_O_4_, Inorganic chemistry, Chemical synthesis, Nanoscale materials, Environmental impact

## Abstract

Natural and synthetic zeolites have been considered as capable candidates for removal of pollutants from water and wastewater due to their high surface area and porous structure. In this work, borosilicate zeolite (BZ) with ZSM-5 structure, graphene quantum dots (GQDs)/BZ and Fe_3_O_4_/BZ as two component, and Fe_3_O_4_-GQDs/BZ as three component nanocomposites were prepared using hydrothermal, solid state dispersion, and ultrasound-assisted co-precipitation methods. The prepared samples were characterized by X-ray diffraction (XRD), Fourier transform infrared spectroscopy (FT-IR), field emission scanning electron microscopy (FE-SEM), energy dispersive X-Ray spectroscopy (EDX), N_2_ adsorption-desorption, and vibrating sample magnetometer (VSM) techniques. The synthesized composites were used for the removal of nitrate, methylene blue (MB), 4-nitrophenol (4-NPh), biological oxygen demand (BOD), and chemical oxygen demand (COD) from water and industrial wastewater. Among the studied adsorbents, the three-component nanocomposites demonstrated superior performance in removing nitrate and cationic and anionic dyes, achieving removal efficiencies of 85.5% for nitrate, 98.4% for MB, 91.0% for 4-NPh, 100.0% for BOD, and 60.0% for COD. The kinetics studies revealed that all adsorbents obey the pseudo-second-order kinetic model. The prepared new multifunctional magnetic nanocomposites composed of graphene quantum dots is expected to be promising adsorbents for the removal of pollutants from water and wastewater.

**Supplementary Information:**

The online version contains supplementary material available at 10.1038/s41598-025-07746-4.

## Introduction

Nowadays, the removal of water contaminants, including hazardous chemicals, dyes, heavy metal ions, pharmaceuticals, bacteria, etc., introduced into water from different sources is of great importance. In recent decades, numerous technologies have been devised for the elimination of these contaminants, with particular emphasis on the use of nanoscale adsorbents^1–4^. Zeolites have been frequently used for the adsorption of a wide variety of pollutants due to their unique porous structure, high surface area, ion-exchange ability, and good thermal and chemical stability^5–8^. The physicochemical characteristics of zeolites can be manipulated by isomorphous substitution of heteroatoms in their framework. This procedure involves replacing the framework aluminum with heteroatom, which typically occupies a tetrahedral position^9–11^. Isomorphic substitution creates novel variant of the zeolites maintaining the initial structure, potentially applicable in emerging domains^12^. Despite the great efficiency of zeolites and other adsorbents, the separation of these materials after pollutant removal still remains as a challenge. The instrumental methods for separating adsorbents from water and wastewater are not economically feasible. Magnetic adsorption technique has been proposed as a novel technology to this drawback, due to its great efficiency and simplicity of separation after treatment. Magnetic nanocomposites are among the most important and extensively utilized categories of nanomaterials for environmental remediation^13–15^. In this regards, Oliveira et al.^16^ reported the synthesis of NaY zeolite with iron oxide composites for the removal of metallic contaminants (Cr^3+^, Cu^2+^ and Zn^2+^) from water. The adsorption features of zeolites were combined with magnetic properties of iron oxides in the prepared composites and the obtained materials showed high adsorption capacities for all tested metal ions. AbdulRazak and co-workers^17^ studied the capability of a magnetic composite of zeolite 13X with Fe_3_O_4_ in the removing anionic Biebrich Scarlet dye from aqueous solutions. They used Response Surface Method to predict the performance of composite in dye removal and obtain the optimal condition. According to the results, the experimental values were in good agreement with the predicted ones.

We synthesized Fe_3_O_4_/Bentonite nanocomposite using chemical co-precipitation and sonochemical methods^18^. The prepared nanocomposite could efficiently remove nitrate from water (about 79%) and decrease the biological oxygen demand (BOD) and chemical oxygen demand (COD) of pharmaceutical wastewater up to 85% and 89%, respectively. The magnetic nanocomposite was readily separated from the liquid phase by a magnet. More examples on the synthesis of magnetic zeolites and their applications, especially in water treatment, are available in the literature^19,20^.

Carbon nanostructures, including carbon quantum dots and graphene, have garnered significant interest for water treatment due to their distinctive chemical and electrical properties, as well as their potential for functionalization with mineral nanomaterials. Graphene quantum dots (GQDs) are graphene nanosheets with a thickness of less than 10 nm. Compared to graphene, they benefit from better dispersion in aqueous solutions and typical semiconducting properties^21–23^. It has been claimed that GQDs and their composites can be used for detection and removal of pollutants. Alvand et al.^22^ prepared a core-shell structure Fe_3_O_4_@SiO_2_@GQD nanocomposite and examined its activity for detecting and removing mercury (II) ions. The composite exhibited a linear response to Hg^2+^ in the concentration range of 0.1 to 70 µM under optimum condition. Moreover, the substantial adsorption capacity for Hg^2+^ (68 mg g^−1^) was attributed to the presence of GQDs possessing a high specific surface area and various functional groups. Hydrothermal synthesis of a graphene/magnetite/montmorillonite nanocomposite was reported by Zhang et al.^24^. The nanocomposite was used for the removal of MB from water and the effect of different parameters including pH, temperature, contact time, dye concentration and ultrasonication on the adsorption capacity was studied. The results confirmed the excellent potential of nanocomposite for removing the cationic dye MB. GQD magnetic nanocomposites have also been investigated for the removal of phenolic compounds^21^ elimination of Pb (II) ions from water^25^ analysis of food contaminants^26^ catalytic reduction of nitrophenol^27^ etc. Considering the efficiency of magnetic nanocomposites in water treatment, we aimed the synthesis of three-component composites comprised of GQDs, magnetic Fe_3_O_4_ nanoparticles and borosilicate zeolite in this work. Borosilicate zeolites are obtained by replacing aluminum atoms with boron atoms in the zeolite framework^12^. They can be used as supports in the preparation of nanocomposites for extensive applications, including water and wastewater pollutant purification processes. Furthermore, the modification of zeolites with GQDs results in improved versions of zeolite catalysts characterized by high surface area and superior adsorption capabilities^24,28^. The synthesis procedure was conducted in an ultrasound bath. By application of ultrasound waves, a number of small bubbles are formed in the solution which their subsequent explosion releases large amounts of energy and provides suitable condition for most of chemical reactions, especially the synthesis of nanoparticles^29,30^. Ultrasonic bath can help the mixing and homogenization of suspension and have a significant effect on particle size reduction, as proven in our previous work^18^.

The development of three-component hybrid composites using borosilicate zeolite as a support establishes an emerging domain within the field of graphene quantum dot-functionalized magnetic zeolites, a pioneering endeavor undertaken in this study. Furthermore, in the synthesis of the aforementioned nanomaterials, efforts have been made to employ procedures that are industrially applicable, biocompatible, and cost-effective.

## Experimental

### Materials

Citric acid monohydrate (C_6_H_8_O_7_.H_2_O), ethanol (96%), ammonia solution (25%), and sulfuric acid (98%) were purchased from Dr. Mojallali chemical industries complex (Tehran, Iran). Iron(II) chloride tetrahydrate (FeCl_2_.4H_2_O), iron(III) chloride hexahydrate (FeCl_3_.6H_2_O), Iron(III) nitrate nonahydrate (Fe(NO_3_)_3_.9H_2_O), potassium nitrate (KNO_3_), potassium dichromate (K_2_Cr_2_O_7_), silver sulfate (Ag_2_SO_4_), mercury(II) sulfate (HgSO_4_), 4-nitrophenol (4-NPh), and methylene blue (MB) were procured from Merck company. All chemicals were used as received.

### Synthesis of GQDs/borosilicate nanocomposite

To fabricate GQDs/borosilicate (GQD/BZ) composite, MFI-type borosilicate zeolite (BZ) and GQDs were respectively synthesized via the hydrothermal method and pyrolysis of citric acid, as described in our previous works^12,28^. The obtained materials were mixed together in a specified weight ratio (GQD: BZ ratio of 1:10) and the mixture was dispersed in 15 mL of ethanol and subjected to ultrasound waves for 3 h to obtain a homogeneous suspension. Subsequently, it was stirred with a magnetic stirrer for 48 h in a capped container. The lid was then removed, and the ethanol was evaporated at room temperature. Finally, the composite was dried at 100 °C overnight.

### Synthesis of Fe_3_O_4_-borosilicate nanocomposites

Fe_3_O_4_-borosilicate magnetic nanocomposites with different weight percentages (20 and 50 wt%) of iron oxide were prepared by co-precipitation/sonication method. To this end, a solution of iron salts, including iron(II) chloride and iron(III) chloride were dissolved in distilled water at 25 °C. Subsequently, 1 g of the prepared borosilicate zeolite was added to it and completely dispersed within the solution using an ultrasound bath (Sonicator 2200 MH S3, operating at 40 kHz ± 5% with a maximum output power of 305 W). Then, specific amounts of ammonia solution were added dropwise to the resulting suspension under nitrogen atmosphere until pH = 10. The stirring was continued for 1 h at 80 °C under nitrogen atmosphere. Finally, the magnetic nanocomposite was centrifuged, washed several times with distilled water and dried at room temperature. The nanocomposites prepared in this way were designated as Fe_3_O_4_ x%-BZ (x = 20 and 50).

### Synthesis of Fe_3_O_4_-GQDs/borosilicate nanocomposites

The three-component nanocomposites of Fe_3_O_4_-GQDs/borosilicate were prepared using the same method described in Sect.2.3 for synthesis of Fe_3_O_4_-borosilicate composites. For this purpose, GQDs/BZ nanocomposite (prepared according to Sect.2.2) was used as the support instead of borosilicate zeolite. The resultant composites containing different weight percentages of Fe_3_O_4_ were denoted as Fe_3_O_4_ x%-GQD/BZ (x = 20 and 50).

### Characterization apparatus

X-ray diffraction patterns (XRD) were collected by a Tongda TD-3700 diffractometer using Cu kα radiation (λ = 1.5406 Aº and 2θ = 4–70°) at room temperature. Fourier transform infrared spectra (FT-IR) was recorded using KBr pellets by a Bruker Tensor 27 instrument in the range of 400–4000 cm^−1^. Scanning electron microscope (Tescan MIRA3 FE-SEM, operating at 15 kV) equipped with energy dispersive X-ray facility (EDX, accelerating voltage equal to 20 kV) was used to provide SEM images and perform elemental analysis. Brunauer–Emmett–Teller (BET) surface area and pore size distribution of composites were calculated by analyzing the N_2_ adsorption–desorption isotherms acquired using a Micromeritics instrument at liquid nitrogen temperature. Magnetic properties of the samples were investigated using a vibrating sample magnetometer (VSM) at room temperature.

### Adsorption/removal tests

The capability of prepared materials was evaluated via adsorption and removal of different contaminants. Nitrate, 4-nitrophenol and methylene blue, which are present in the waste of most industries, were chosen as representative pollutants. The tests were conducted under optimum conditions determined in our previous works^18,31–33^. Furthermore, the effectiveness of composites in reducing BOD and COD values of real samples was investigated.

#### Removal of nitrate

A stock solution was prepared by dissolving 1.63 g potassium nitrate in 1 L distilled water and used for preparation of standard solutions. The light absorbance of standard solutions was measured employing a UV-vis spectrophotometer (Shimadzu MPC-2200 UV spectrophotometer) at λ_max_ = 205 nm and the plotted calibration diagram was applied for determination of nitrate concentration after adsorption tests^34^. In each adsorption test, 50 mL of nitrate solution (30 ppm) was stirred with 0.2 g of adsorbent for 2 h. The pH of solution has been adjusted to 4. The adsorbent was then separated from solution and the removal efficiency was calculated using Eq. (1).

1 $$\text{Re} moval\,efficiency~\left( \% \right) = ~\frac{{C_{0} - ~C_{t} }}{{C_{0} }}~ \times 100$$

where C_0_ and C_t_ stand for initial concentration and concentration at time t, respectively^35^.

The removal tests were repeated three times for each adsorbent and the average values were reported as final data.

To further confirm the results of removal tests obtained by UV spectrophotometer, ion chromatography method was also used in some cases.

#### Photocatalytic removal of 4-NPh and MB

For evaluating the photocatalytic removal of organic pollutants, a particular amount of nanocomposite (0.05 g for 4-NPh and 0.1 g for MB removal) was added to 50 mL of pollutant solution (20 ppm). The mixture was stirred for 30 min in the dark using a mechanical stirrer (130–200 rpm) to achieve an adsorption/desorption equilibrium. Then, the suspension was exposed to visible light and the samples were taken at every 30 min for 180 min. A typical 36 W cool white fluorescent lamp, located above the reaction medium was used as visible light source. The samples were centrifuged and the concentration of pollutant in supernatant was determined by measuring the absorbance at λ_max_ (315 nm for 4-NPh and 668 nm for MB). In the case of 4-NPh, one droplet of concentrated sulfuric acid was added to the supernatants before measuring the light absorbance^31,33^. Notably, 4-NPh exists in equilibrium between its phenol and phenolate forms at neutral pH (4-NPh has a pK_a_ of 7.15 at 25 °C). The acid is added to ensure the complete protonation of the phenolate ions to the phenol form. Then the light absorbance is measured at 315 nm which is the λ_max_ of the protonated form of 4-NPh. The removal efficiency is estimated using Eq. ().

#### BOD and COD removal from industrial wastewater

BOD and COD are two important factors typically assessed to evaluate the degree of water pollution^36^. BOD is a measure of oxygen consumed by microorganisms in water for degradation of organic materials, whereas COD measures the quantity of required oxygen for chemical oxidation of total organic and inorganic pollutants in water^37^. To explore the effect of prepared nanocomposites in BOD reduction, 0.1 g of each nanocomposite and 50 mL industrial wastewater were mixed together in special BOD digital bottles. The bottles were transferred to the incubator at 20 °C and kept for five days. After that the BOD_5_ was measured and the efficiency was calculated using the initial and final values of BOD. The COD was measured by the open reflux dichromate titrimetric method. For this purpose, a digestion solution was prepared by mixing 20 mL potassium dichromate solution and 30 mL concentrated sulfuric acid containing silver sulfate as catalyst. A desired amount of mercury(II) sulfate was also added to the solution to eliminate interferences. Then 0.3 g of nanocomposite was added into 50 mL industrial wastewater (initial COD ≈ 5000) and the suspension was stirred for 2 h at room temperature. Afterwards, the suspension was poured into digestion solution and refluxed for 2 h at 150 °C. The mixture was cooled to room temperature and the COD was determined by titrating the mixture with iron(III) nitrate solution^34^.

## Results and discussion

### Characterization

**Fig. 1:**
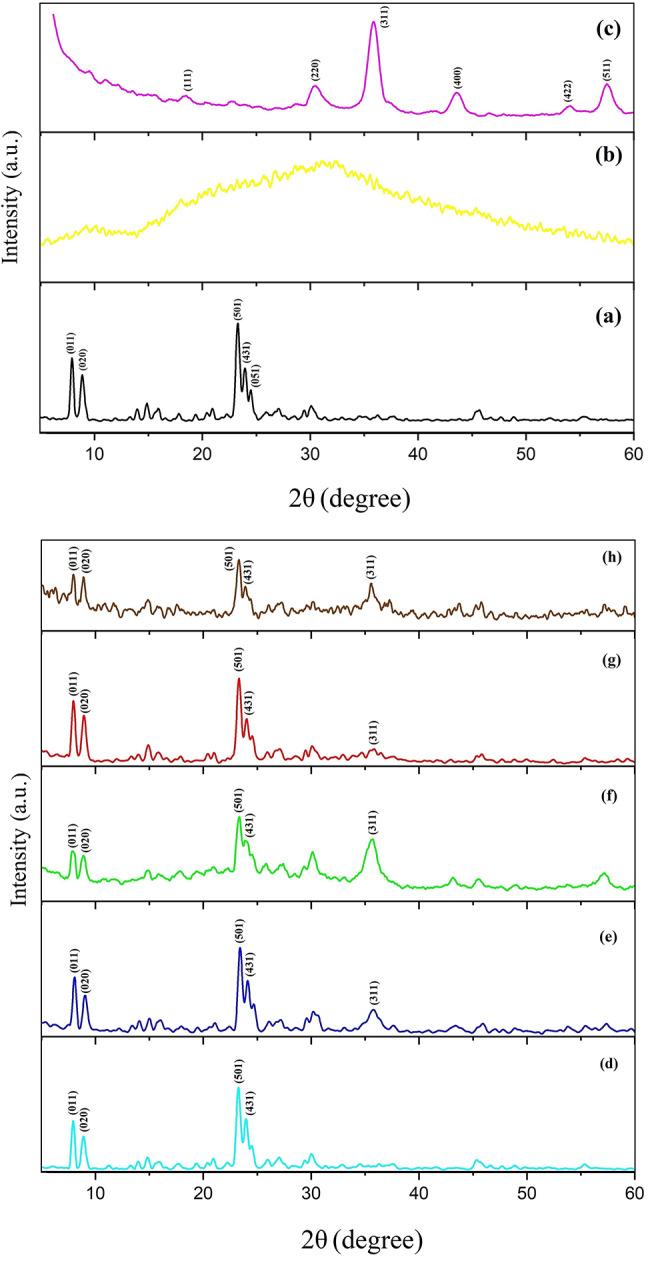
XRD patterns of borosilicate (**a**), GQDs (**b**), Fe_3_O_4_ (**c**), GQD/BZ (**d**), Fe_3_O_4_20%-BZ (**e**), Fe_3_O_4_50%-BZ (**f**), Fe_3_O_4_20%-GQD/BZ (**g**), and Fe_3_O_4_50%-GQD/BZ (**h**).

The indexed XRD patterns of prepared nanocomposites are presented in Fig. 1. For a precise comparison, the XRD patterns of pure constituents of nanocomposites (borosilicate zeolite, GQDs, and Fe_3_O_4_ nanoparticles) are also depicted in Fig. 1a-c. The XRD pattern of borosilicate (Fig. 1a) shows the characteristic peaks of MFI structure in the 2θ range of 7–10 ° and 23–25 ° which confirms the synthesis of pure single phase zeolite^38^. On the other hand, the XRD pattern of GQDs shown in Fig. 1b, presents a relatively broad characteristic peak at 2θ ≈ 20–40 ° corresponding to the (002) plane in carbon materials^39^. The broadening of this peak can be considered as an evidence for the formation of very small crystallites in GQDs. No other distinct peaks are seen in the XRD pattern of GQDs, indicating that the prepared sample is pure. A comparison between the XRD pattern of Fe_3_O_4_ nanoparticles (Fig. 1c) and magnetite phase reference pattern (JCPDS card No. 75–0449) reveals the successful synthesis of highly crystalline Fe_3_O_4_. The most intense peak of magnetite structure appears at 2θ = 36 ° which is related to the (311) reflection plane and proves the formation of spinel phase. Besides, the peaks of other impurities such as the goethite phase are not seen in the XRD pattern of Fe_3_O_4_ nanoparticles. It is worth noting since the XRD pattern of the magnetite and maghemite (γ-Fe_2_O_3_) phases are alike, the prepared sample may contain a small amount of the maghemite phase, but what is certain is that due to the high magnetic property of the nanoparticles, the non-magnetic phases are absent or present in negligible amounts in the sample. The XRD pattern of GQDs/borosilicate composite is shown in Fig. 1d. As can be seen, the XRD pattern of this composite is similar to that of pure borosilicate zeolite, except that the intensity of the borosilicate peaks has decreased. The characteristic peak of GQDs is not observed in the XRD pattern due to its low proportion in composite. In contrast, the main peak of Fe_3_O_4_ at 2θ ≈ 36 ° appears in the XRD patterns of Fe_3_O_4_-BZ composites with different contents of iron oxide (Fig. 1e, f). This peak can also be detected in the XRD patterns of Fe_3_O_4_-GQD/BZ nanocomposites (Fig. 1g, h). The intensity of mentioned peak increases with increasing the content of Fe_3_O_4_ in nanocomposites, while the intensity of borosilicate peaks decreases. The appearance of characteristic peaks of borosilicate zeolite indicates the stability of the zeolite crystal structure during the synthesis processes.

**Fig. 2:**
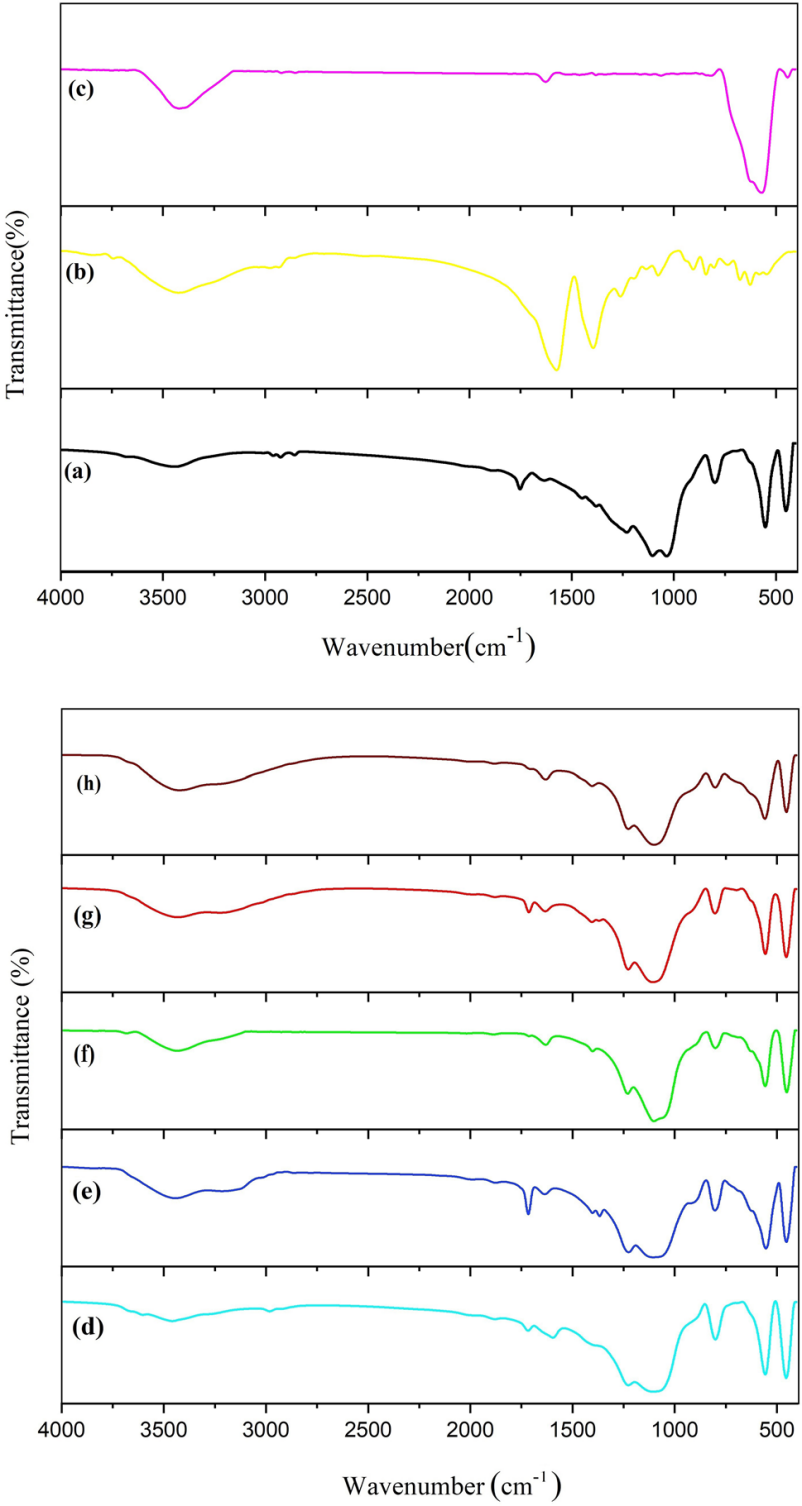
FT-IR spectra of borosilicate (**a**), GQDs (**b**), Fe_3_O_4_ (**c**), GQD/BZ (**d**), Fe_3_O_4_20%-BZ (**e**), Fe_3_O_4_50%-BZ (**f**), Fe_3_O_4_20%-GQD/BZ (**g**), and Fe_3_O_4_50%-GQD/BZ (**h**).

Figure 2 presents the FT-IR spectra of prepared materials. The FT-IR spectrum of borosilicate zeolite (Fig. 2a) shows a characteristic band at 551 cm^−1^ which is assigned to the vibrations of 5-membered double rings and confirms the formation of MFI structure. Besides, the incorporation of boron into zeolite framework is approved by the appearance of a doublet band at 1000–1100 cm^−1^ corresponded to the internal asymmetric stretching vibrations of O – T – O (T = Si and B). The bands at 450 and 798 cm^−1^ are attributed to the bending vibration and external symmetric stretching vibrations of T – O – T, respectively^40^. In the FT-IR spectrum of GQDs (Fig. 2b), characteristic bands assignable to the C – H bending vibration, C – OH stretching vibration, symmetric stretching vibration of carboxyl groups, and aromatic C = C vibration can be observed at 841, 1075, 1393, and 1572 cm^−1^, respectively. Furthermore, a shoulder appears around 1718 cm^−1^ which can be attributed to the C = O stretching vibration of the carboxyl groups^28,41^. The FT-IR spectrum of Fe_3_O_4_ (Fig. 2c) shows a weak band at 443 cm^−1^ and a broad and sharp band at 569 cm^−1^ due to the vibration of Fe – O bonds in octahedral and tetrahedral sites of spinel phase, respectively. A broad band appears at about 3400 cm^−1^ in the FT-IR spectra of all samples, which is attributed to the stretching vibration of hydroxyl groups^42^.

The FT-IR spectra of all composites are similar to that of pure borosilicate, except for a few small changes as following: (i) For GQD/BZ nanocomposite (Fig. 2d), two bands appear at 1400 and 1594 cm^−1^ due to the presence of GQDs. Because of GQDs low content in nanocomposite, the bands have low intensity. On the other hand, the bands are shifted to higher wavenumbers in comparison to pure GQDs, which could originate from the strong interactions between GQDs and borosilicate in this composite. The band at 1400 cm^−1^ is observable in the FT-IR spectra of three-component composites, as well (Fig. 2f, g). (ii) The characteristic band of zeolite at 551 cm^−1^ is broaden in the FT-IR spectra of composites with different amounts of Fe_3_O_4_ (Fig. 2e-h). This can be resulted from the overlap of this band with the strong band of Fe_3_O_4_ in this region.

**Fig. 3:**
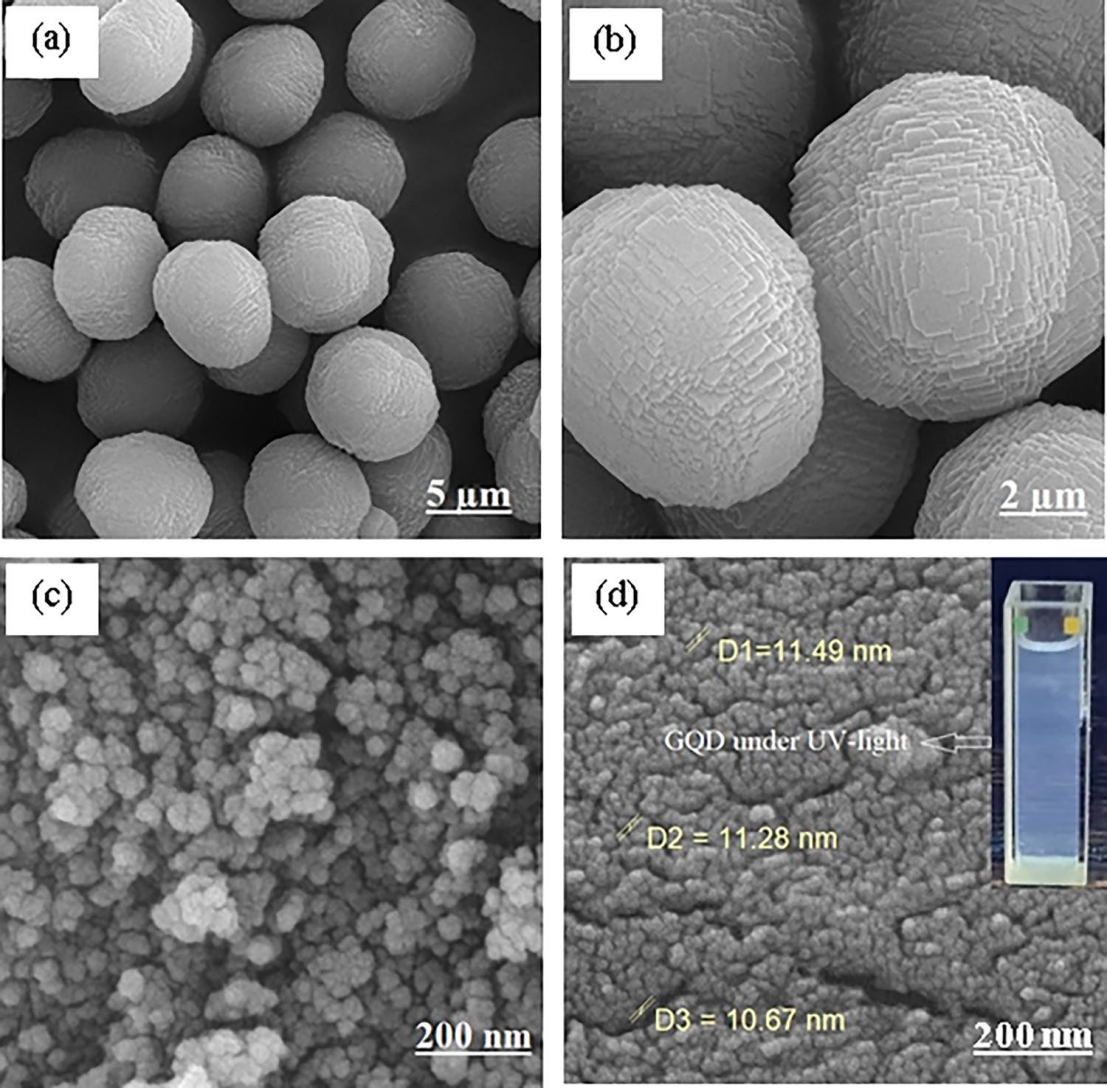
FE-SEM images of borosilicate (**a**, **b**), Fe_3_O_4_ (**c**), and GQDs (**d**). Inset shows the emission of GQDs under UV lamp.

**Fig. 4:**
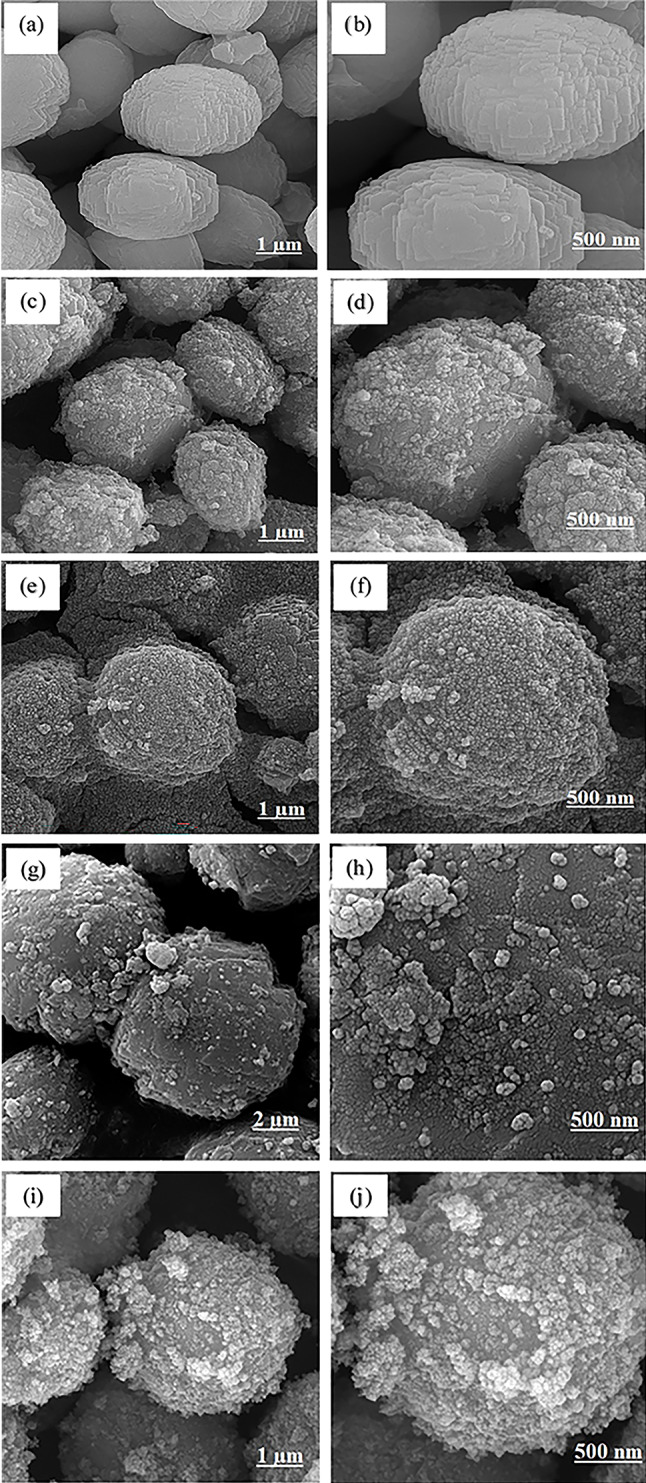
FE-SEM images of GQD/BZ (**a**, **b**), Fe_3_O_4_20%-BZ (**c**, **d**), Fe_3_O_4_50%-BZ (**e**, **f**), Fe_3_O_4_20%-GQD/BZ (**g**, **h**), and Fe_3_O_4_50%-GQD/BZ (**i**, **j**) nanocomposites.

**Fig. 5:**
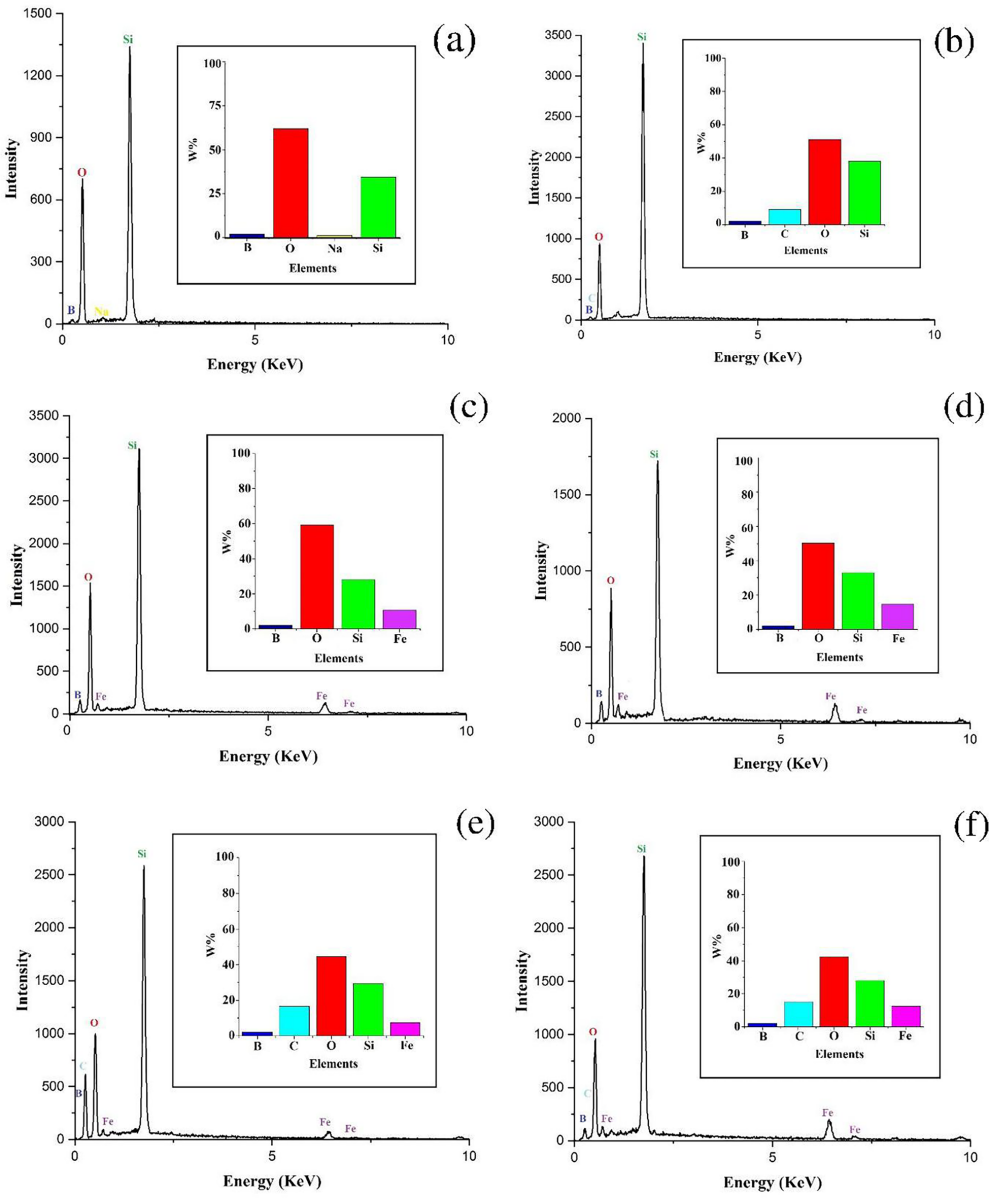
EDX spectra of borosilicate zeolite (**a**), GQD/BZ (**b**), Fe_3_O_4_20%-BZ (**c**), Fe_3_O_4_50%-BZ (**d**), Fe_3_O_4_20%-GQD/BZ (**e**), and Fe_3_O_4_50%-GQD/BZ(**f**). Insets show the weight percentages of elements.

The morphology and elemental composition of prepared materials were studied using the field emission scanning electron microscopy (FE-SEM) and energy dispersive X-ray spectroscopy (EDX). FE-SEM images of borosilicate zeolite (Fig. 3a, b) show the micron-sized spheres composed of plate-like particles. The peaks related to Si, B, O, and Na appear in the EDX spectrum of zeolite (Fig. 4a). Spherical nanoparticles can be seen in the FE-SEM image of pure Fe_3_O_4_ (Fig. 3c). The nanoparticles agglomerated due to their magnetic property. The GQDs have spherical morphology with small dimensions in accordance with XRD results. The small size of GQDs is also established by their blue emission under UV light (inset in Fig. 3d). The FE-SEM images of nanocomposites are presented in Fig. 5. The morphology of borosilicate zeolite does not change significantly in GQDs/BZ nanocomposite owing to the low content of GQDs. The presence of GQDs in nanocomposite and their uniform distribution is proved by appearance of C peak in EDX spectrum (Fig. 4b) and its uniform dispersion in mapping images (Fig. S1). On the other hand, the Fe_3_O_4_ nanoparticles can be observed on the surface of borosilicate in Fe_3_O_4_-BZ and Fe_3_O_4_-GQD/BZ nanocomposites. The distribution of nanoparticles in Fe_3_O_4_-BZ nanocomposites is more uniform than three-component composites. It seems that the presence of GQDs in GQD/BZ support hinders the uniform formation of magnetite nanoparticles on zeolite surface which leads to the aggregation of Fe_3_O_4_ nanoparticles. By increasing the content of Fe_3_O_4_ in nanocomposites, their amount increases on the surface; so that the surface of borosilicate is thoroughly covered by nanoparticles in Fe_3_O_4_ 50%-BZ. The intensity of Fe peak in EDX spectra increases by incremental loading of Fe_3_O_4_ in nanocomposites (Fig. 4).

**Fig. 6:**
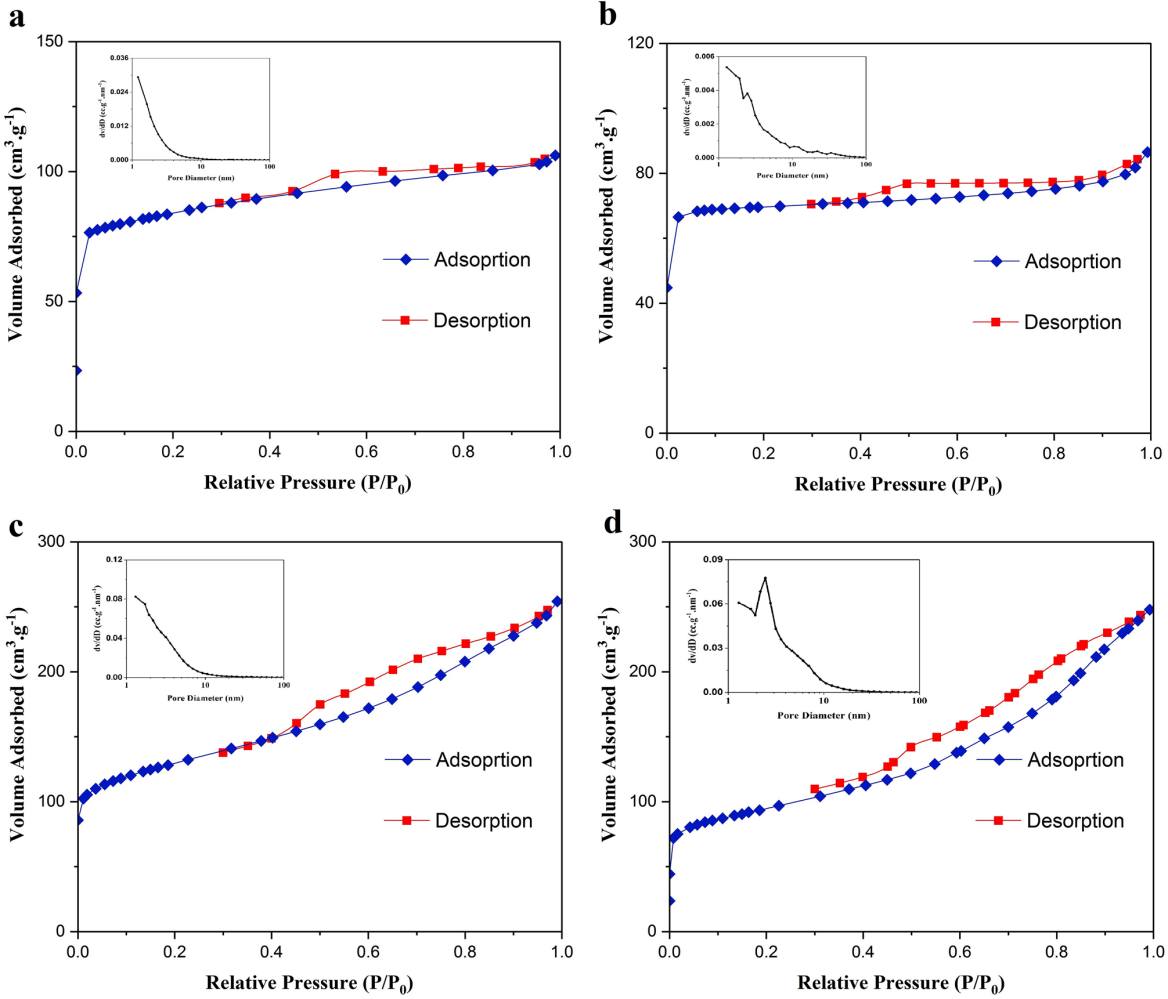
N_2_ adsorption-desorption isotherm and pore size distribution of borosilicate (**a**), GQD/BZ (**b**), Fe_3_O_4_20%-GQD/BZ (**c**), and Fe_3_O_4_50%-GQD/BZ (**d**).

The N_2_ adsorption-desorption isotherm and pore size distribution of borosilicate zeolite and corresponding composites are illustrated in Fig. 6. For all samples a combination of type I and type IV isotherm with a H4 hysteresis loop is observed, which confirms the co-existence of micropores and mesopores. The comparison of surface areas calculated by BET method (Table 1) shows the surface area decreases by preparation of GQD/BZ composite. Such a reduction was observed for composites of GQDs with other metalosilicate zeolites due to the limiting of surface by GQDs which decreases the opportunity for adsorption of N_2_ molecules^28^.

By synthesis of Fe_3_O_4_ on GQD/BZ, the surface area increases and three-component composites have higher BET areas than bare borosilicate and GQDs/BZ nanocomposite. Formation of a porous structure during preparation of Fe_3_O_4_ nanoparticles which is evident from the increased micropore and mesopore volumes could result in high surface areas^43^. By loading higher amounts of Fe_3_O_4_ in Fe_3_O_4_50%-GQD/BZ composite, the surface area and pore volume decreases. The observed decreases probably originate from the aggregation of magnetic nanoparticles at higher content. Pore size distribution of prepared materials was calculated using Barrett-Joyner-Halenda (BJH) method. The BJH curves (presented as insets in Fig. 6) show the maximum peak of pore size distribution shifts to higher values in Fe_3_O_4_50%-GQD/BZ nanocomposite due to the presence of more mesopores in this sample.

**Table 1:** Textural properties of prepared samples.

Sample	a_s, BET_ (m^2^g^−1^)	V_t_^a^ (cm^3^g^−1^)	V_micro_^b^ (cm^3^g^−1^)	V_meso_^c^ (cm^3^g^−1^)	Average pore diameter ^a^ [nm]
Borosilicate zeolite	320.7	0.164	0.146	0.018	2.0
GQD/BZ	271.6	0.133	0.109	0.024	2.0
Fe_3_O_4_20%-GQD/BZ	464.0	0.393	0.306	0.087	3.4
Fe_3_O_4_50%-GQD/BZ	337.9	0.382	0.264	0.118	4.5

^a^Calculated from BET plot.

^b^ Obtained from t-plot.

^c^V_meso_=V_t_ - V_micro_.

**Fig. 7:**
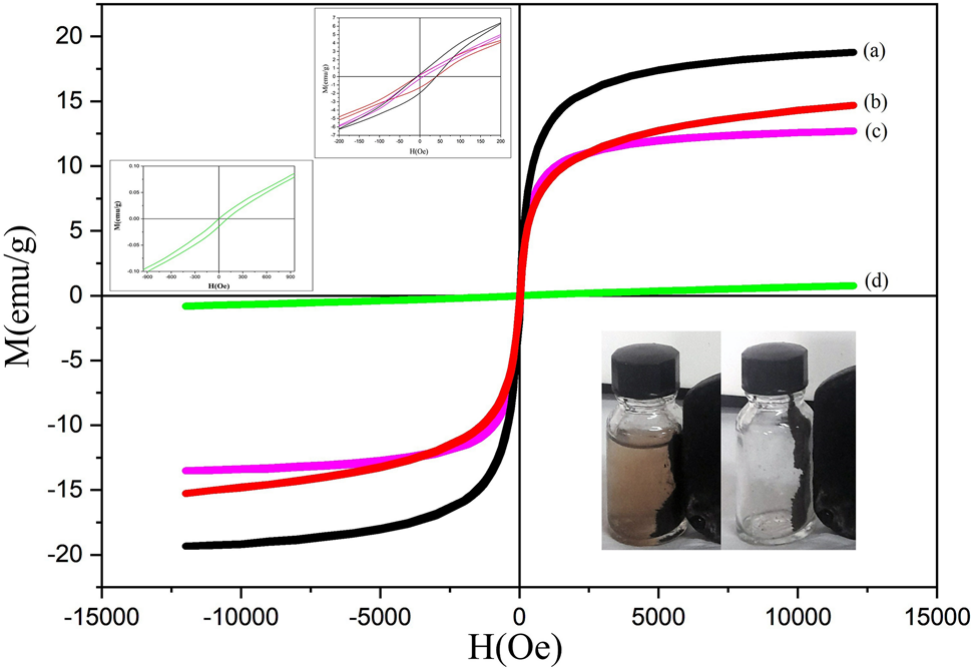
M-H curves of Fe_3_O_4_50%-BZ (**a**), Fe_3_O_4_50%-GQD/BZ (**b**), Fe_3_O_4_20%-BZ (**c**), and Fe_3_O_4_20%-GQD/BZ (**d**). (Left inset: hysteresis loops in expanded scale. Right inset: the photographs of applying an external magnetic field on Fe_3_O_4_50%-GQD/BZ powder and its suspension in water.).

The magnetic feature of composites was assessed by VSM analysis at room temperature and the M-H curves are presented in Fig. 7. For comparison, the M-H curve of pure Fe_3_O_4_ nanoparticles is provided in Fig. S2. Saturation magnetization (Ms), remanent magnetization (Mr), and coercivity (Hc) values, extracted from M-H curves are summarized in Table 2. The appearance of narrow S-shaped hysteresis loops for all samples reveals the ferromagnetic character of prepared composites^44^. Accordingly, the nanocomposites have small coercivity and remanent magnetization which is typical for soft magnetic materials. Such a magnetic material can be easily magnetized by applying a small external magnetic field and quickly demagnetized by omitting magnetic field^44,45^. As expected, by decreasing the magnetic phase in nanocomposites, the saturation magnetization decreases.

Moreover, the Ms values are lower for Fe_3_O_4_-GQD/BZ composites in comparison to Fe_3_O_4_/BZ composites with the same Fe_3_O_4_ content. A similar decrease in Ms value as a result of GQDs presence was reported for GQDs/Fe_3_O_4_/MMT by Zhang et al.^24^. The remanent ratio (Rs = Mr/Ms) was found to be lower than 0.5 for all nanocomposites as shown in Table 2, which indicates all samples have multi-domain structures^46^.

The magnetic nature of prepared nanocomposites can be beneficial for their separation from solution. This was tested by applying a magnet on a suspension of Fe_3_O_4_50%-GQD/BZ composite in water. The photograph in Fig. 7 (right inset) shows that although the saturation magnetization of nanocomposites is lower than that of pure Fe_3_O_4_, they can be readily separated from the solution by a magnet.

**Table 2:** Ms, mr, hc, and Rs parameters of nanomaterials.

Sample	M_s_ (emu/g)	H_c_ (Oe)	M_*r*_ (emu/g)	*R*_s_
Fe_3_O_4_50%-BZ	18.77	6.41	0.29	0.015
Fe_3_O_4_50%-GQD/BZ	14.68	5.18	0.18	0.012
Fe_3_O_4_20%-BZ	12.70	5.94	0.22	0.016
Fe_3_O_4_20%-GQD/BZ	0.78	0	0	0
Fe_3_O_4_	60.55	3.11	0.41	0.132

**Fig. 8:**
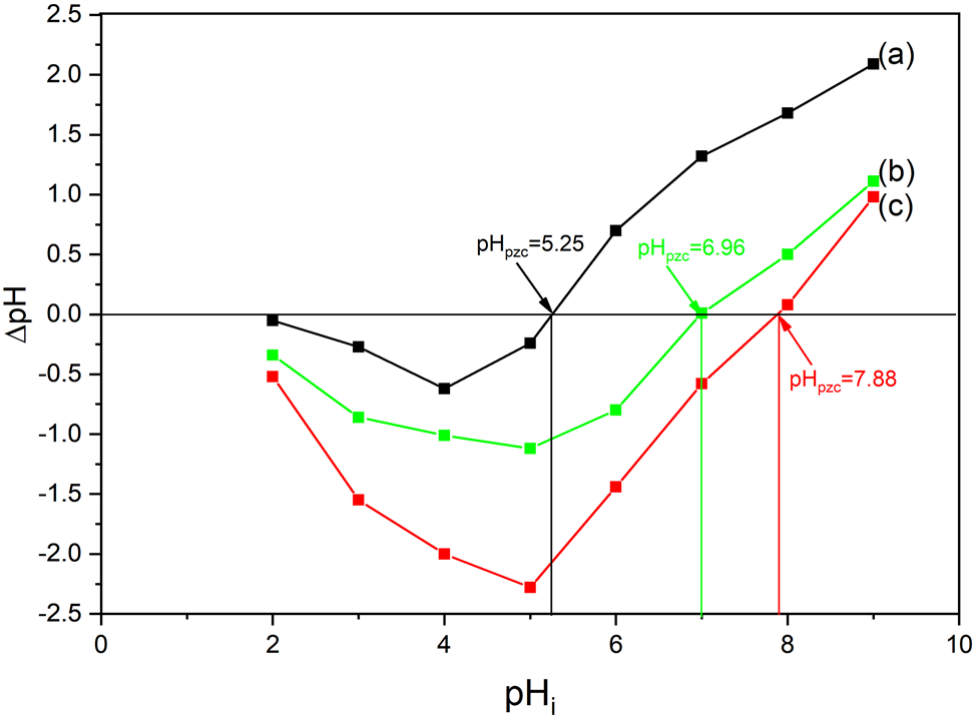
Point of zero charge pH (pH_pzc_) determination for borosilicate (**a**), Fe_3_O_4_20%-GQD/BZ (**b**), and Fe_3_O_4_50%-GQD/BZ (**c**).

The surface charge of the adsorbent is one of the most important factors that can affect its removal efficiency. The surface charge can be determined by estimating the pH of point of zero charge (pH_pzc_). The point of zero charge pH is a pH value in which the surface charge of absorbent is equal to zero. At pH > pH_pzc_, the surface of material has negative charge, whereas at pH < pH_pzc_ the surface is positively charged. By determining pH_pzc_, the appropriate conditions for the adsorption of a pollutant on the adsorbent surface is identified and the adsorption mechanism can be understood. In this work, the pH_pzc_ of bare zeolite and three-component composites were determined using the salt addition method. For this purpose, sodium chloride solutions with initial pH (pH_i_) of 2–9 were prepared and 0.1 g of adsorbent was added to each solution. The suspension was stirred for 24 h at room temperature and then the final pH was measured^47^ The pH_pzc_ was obtained by plotting ∆pH, defined as the difference of initial and final pH (pH_i_ – pH), against pH_i_ (Fig. 8). The value of pH_pzc_ is 5.25, 6.96, and 7.88 for borosilicate zeolite, Fe_3_O_4_20%-GQD/BZ and Fe_3_O_4_50%-GQD/BZ, respectively. The lower pH_pzc_ of borosilicate can be attributed to the acidic nature of zeolites.

### Adsorption/removal studies

####  Adsorption of nitrate from water by prepared nanocomposites

Generally, unmodified zeolites could serve as adsorbents for removal of cations, whereas they are not able to efficiently remove anions because of possible repulsion of their negatively charged structure with anions. The capability of zeolites for removal of anions can be improved through preparation of nanocomposites with various nanoparticles^48^. In this regard, the adsorption of nitrate, a toxic anion, was purposed using synthesized nanocomposites. A batch system was chosen to provide the desired contact between the pollutant and the adsorbent during the adsorption studies. Considering our previous works on optimizing the involved parameters in nitrate adsorption by clay and various zeolites^18,31,49^ the adsorption experiments were carried out using a nitrate solution with initial concentration of 30 ppm and 0.2 g adsorbent. The solution pH was adjusted to 4 which is lower than the pHpzc of adsorbents. pH is one the important factors having essential effect on the adsorption process by affecting the structure of the adsorbate and the surface charge of the adsorbent. At acidic pH, more protons are available which can be added to the adsorbent surface, and the adsorbent surface acquires a positive charge. Therefore, the negatively charged species could be easily adsorbed on the surface via electrostatic interaction^50,51^.

**Fig. 9:**
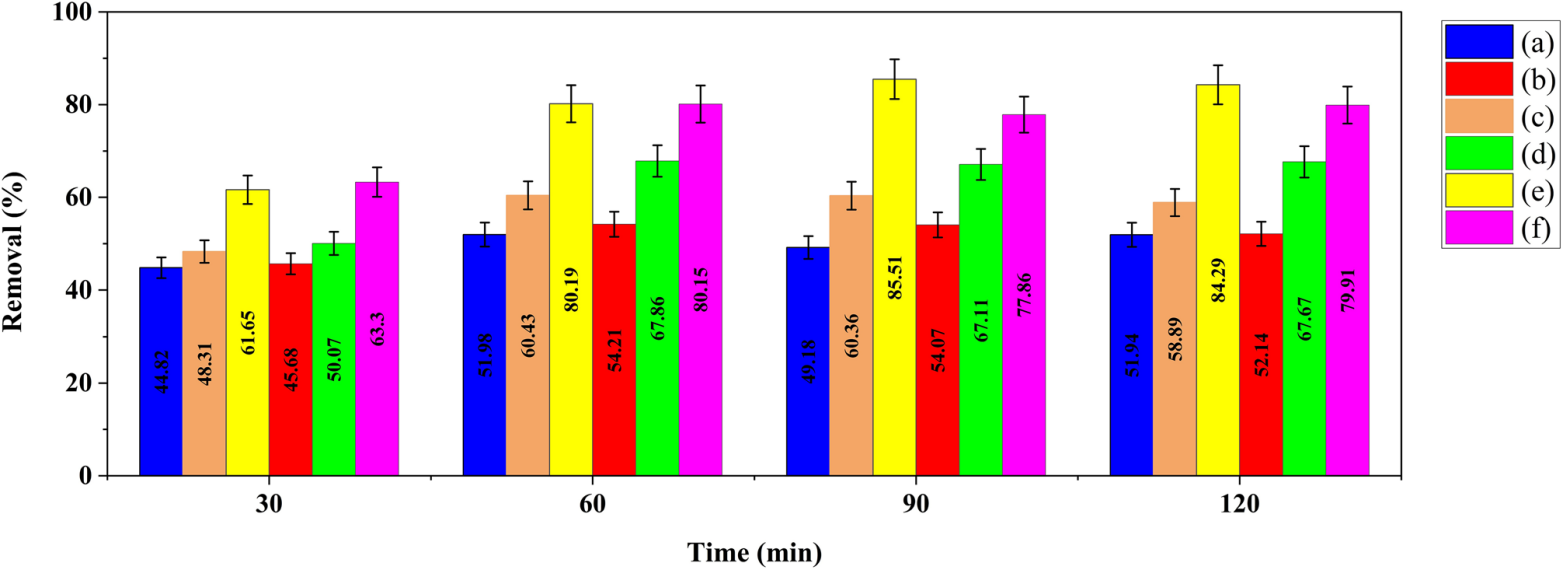
The nitrate adsorption capacity of borosilicate zeolite (**a**), GQD/BZ (**b**), Fe_3_O_4_20%-BZ (**c**), Fe_3_O_4_50%-BZ (**d**), Fe_3_O_4_20%-GQD/BZ (**e**), and Fe_3_O_4_50%-GQD/BZ (**f**).

The nitrate removal capacity of prepared nanocomposites along with pure borosilicate zeolite is displayed in Fig. 9. It is relevant that the major adsorption of nitrate takes place within the first hour and there is no significant change in removal efficiency at longer times. This observation could be due to the fast occupation of adsorption sites by nitrate ions at early times of process which decreases the opportunity for more nitrate adsorption. The active adsorption sites are located in the surface of the adsorbent and as well in balk. At the beginning of the adsorption process, the surface sites directly exposed to nitrate ions, which have a higher likelihood of interaction, become occupied, resulting in a significant rise in adsorption efficiency. However, with the saturation of the surface and external sites, adsorption continues through the internal sites, which will slow down the rate of adsorption due to the mass transfer difficulty^49,52^.

The results indicate that the synthesized nanocomposites present almost similar trends in removal of nitrate; however, the removal efficiency is greater for three-component nanocomposites. This can be attributed to the high surface area of ​​ three-component nanocomposites and dispersion of GQDs and iron oxide nanoparticles on borosilicate zeolite surface, resulting in better interaction of these particles with nitrate. Nitrate adsorption on nanocomposites may occur through Fe atoms on the composite surface interacting with oxygen atoms of nitrate or through hydrogen bonding between O atoms of nitrate ions and surface OH groups of GQDs and Fe_3_O_4_^53^.

Since the aim of this project is water and wastewater treatment, the absorption process was conducted at room temperature, but it is likely that increasing the temperature can increase the activity of nitrate anions, make the surface layer more active, and provide suitable conditions for greater anion adsorption.

**Fig. 10:**
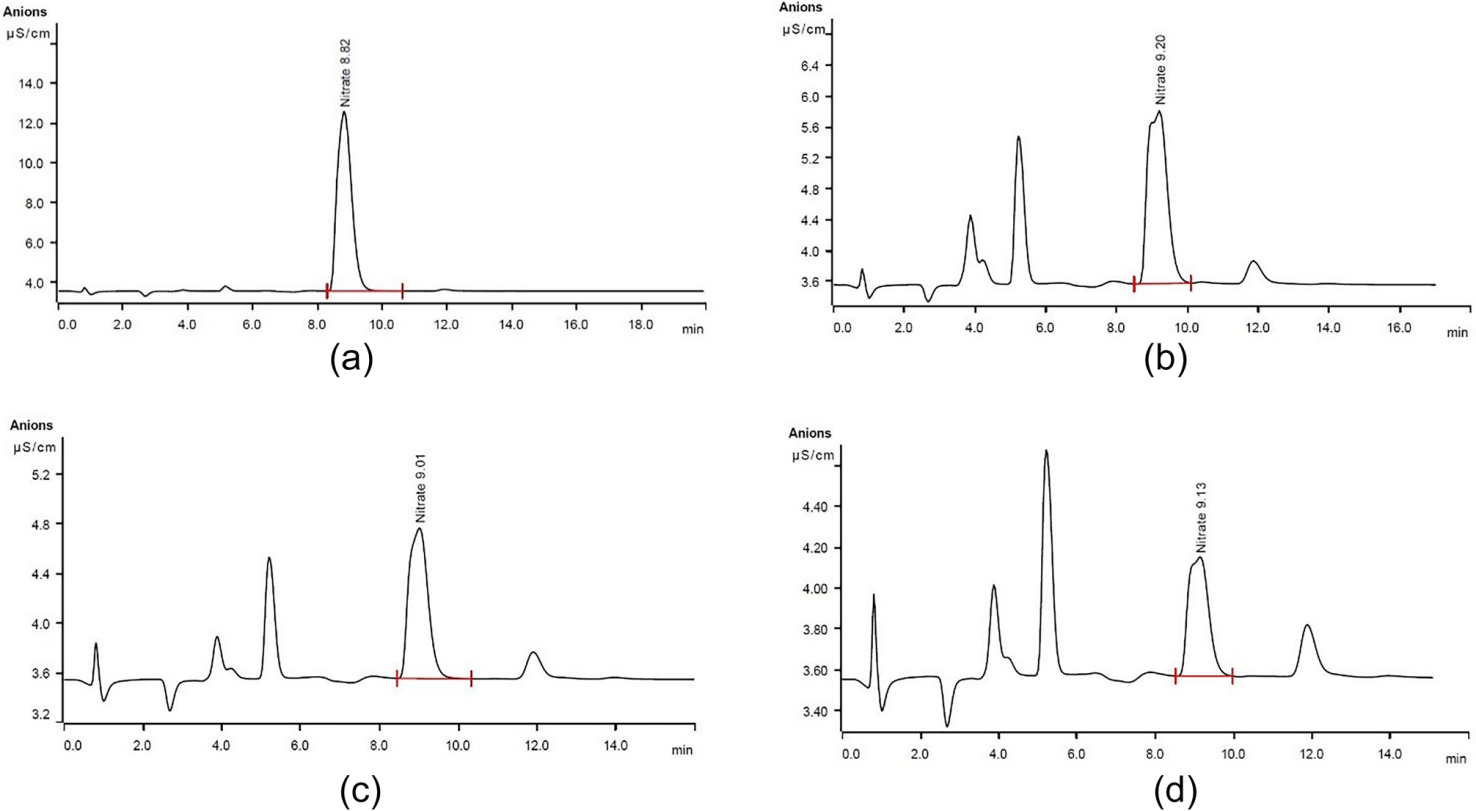
Ion chromatograms of nitrate solution before (**a**), and after adsorption by Fe_3_O_4_50%-BZ (**b**), Fe_3_O_4_50%-GQD/BZ (**c**), and Fe_3_O_4_20%-GQD/BZ (**d**).

The adsorption capacity of some nanocomposite was also investigated using ion chromatography method and the results are shown in Fig. 10; Table 3. As can be seen, the results are consistent with those obtained by UV-vis spectroscopy and confirm the effectiveness of spectrophotometry method for nitrate removal monitoring.

**Table 3:** The parameters extracted from ion chromatograms shown in Fig. 10.

Peak number	Retention time (min)	Area ((µS cm^−1^) min)	Height (µS cm^−1^)	Concentration (ppm)	Removal (%)
a	8.823	4.6532	9.025	27.312	Initial concentration
b	9.202	1.4334	2.239	8.880	67.48
c	9.008	0.6772	1.213	4.551	83.34
d	9.132	0.3506	0.583	2.681	90.19

**Fig. 11:**
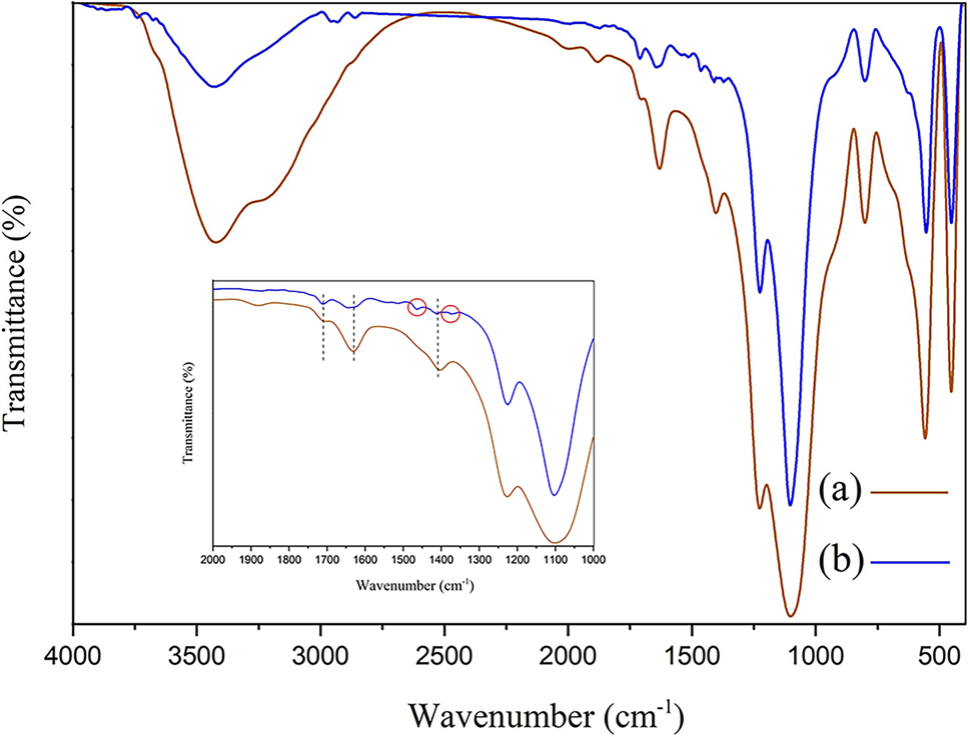
FT-IR spectra of Fe_3_O_4_50%-GQD/BZ nanocomposite before (**a**) and after (**b**) nitrate adsorption. Inset shows the expanded FT-IR spectra in the 1000–2000 cm^−1^.

To further confirm the adsorption of nitrate by nanocomposites, FT-IR spectra of Fe_3_O_4_50%-GQD/BZ after adsorption process was provided and illustrated in Fig. 11. The appearance of bands related to N-O stretching vibration at 1375 cm^−1^ and 1465 cm^−1^ indicates the nitrate adsorption on nanocomposite.

#### Removal of organic pollutants using prepared nanocomposites

In order to evaluate the photocatalytic activity of synthesized materials, the removal of methylene blue, a cationic dye and 4-nitrophenol, a hardly degradable pollutant was investigated under ambient light. The photocatalytic removal of pollutants by photocatalysts took place through two successive stages. In the first step, the pollutant was adsorbed on the surface of adsorbent, and in the next step, the adsorbed pollutants were degraded via reaction with photo-generated reactive oxygen species. The adsorption process played a crucial role in the photocatalytic removal, as it brings the pollutants into contact with reactive oxygen species responsible for photocatalytic degradation which were generated on the surface of photocatalyst under light irradiation. Therefore, the suspensions containing pollutant solution and photocatalyst were initially stirred in the dark for 30 min to achieve complete adsorption of pollutant^28^.

**Fig. 12:**
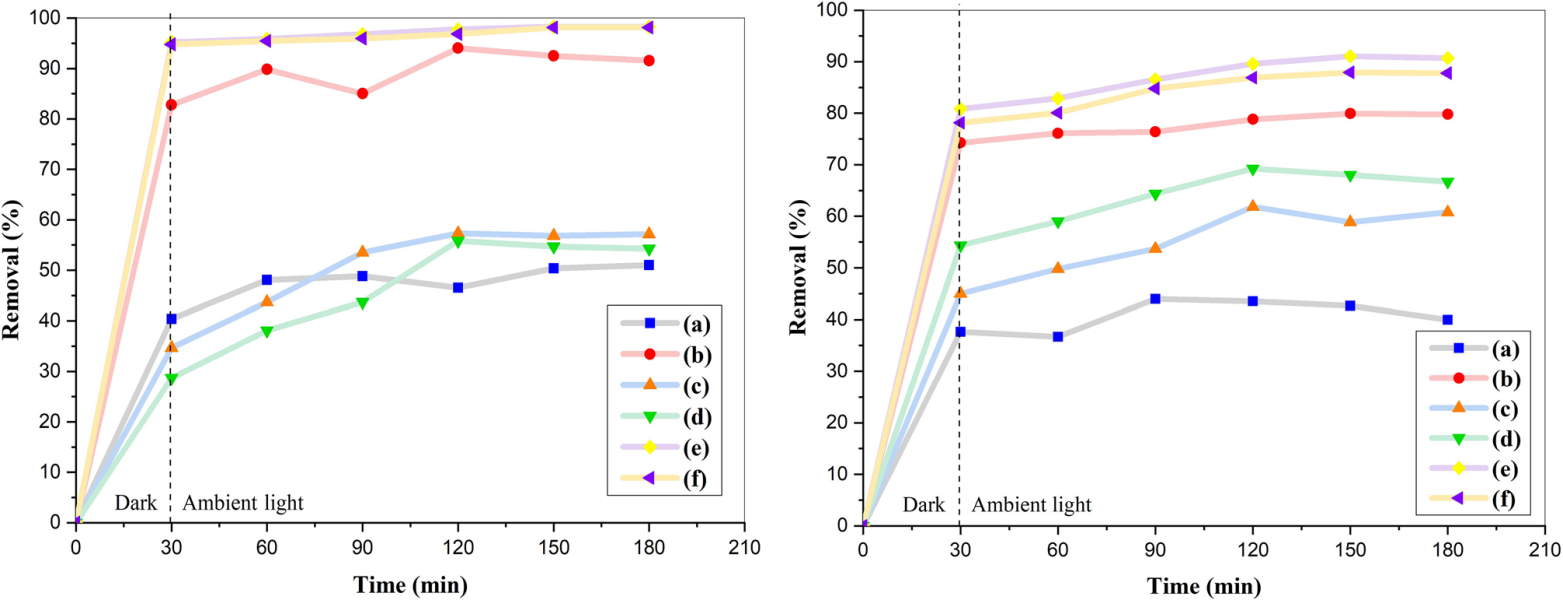
Removal efficiencies of MB (left) and 4-NPh (right) by borosilicate zeolite (**a**), GQD/BZ (**b**), Fe_3_O_4_20%-BZ (**c**), Fe_3_O_4_50%-BZ (**d**), Fe_3_O_4_20%-GQD/BZ (**e**), and Fe_3_O_4_50%-GQD/BZ (**f**).

The UV-vis absorption spectra of MB and 4-NPh solutions before and after removal by prepared materials are shown in Fig. S3 and their removal efficiencies are presented in Fig. 12. As can be seen, a decrease in the light absorption by residual solutions is observed after photocatalytic tests due to the decrement of pollutants concentration. For all samples, the removal efficiency of both pollutants reaches a maximum value after the first 30 min and then changes slightly. Also, the removal percentage of both pollutants by GQD/BZ composite is higher than that of borosilicate zeolite. This observation indicates that while the presence of GQDs on the borosilicate zeolite has led to the decrease in the surface area, their presence, even at low content, could significantly improve the photocatalytic activity of zeolite. On the other hand, the GQD/BZ shows better removal efficiencies than Fe_3_O_4_-BZ composites for both pollutants, which can be attributed to the optical properties of graphene quantum dots. Similar to nitrate removal results, the three-component composites with high surface areas show the highest removal efficiencies for both pollutants.

**Fig. 13:**
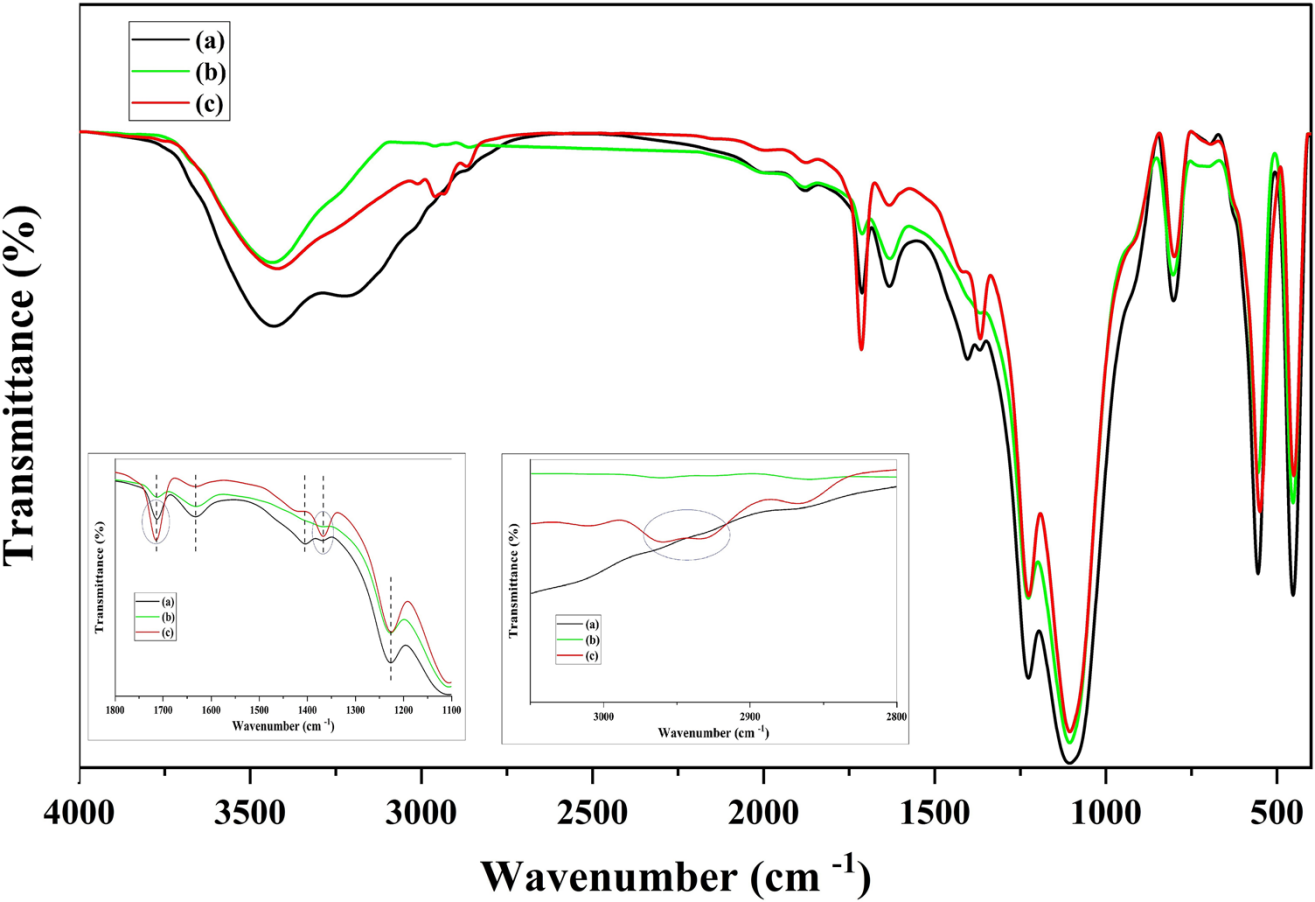
FT-IR spectra of Fe_3_O_4_20%-GQD/BZ composite before (**a**) and after removal of MB (**b**) and 4-NPh (**c**).

The inspection of FT-IR spectra of Fe_3_O_4_20%-GQD/BZ composite before and after MB and 4-NPh adsorption revealed some variations after 4-NPh removal test. As indicated in Fig. 13, two bands appear at 2962 cm^−1^ and 2931 cm^−1^, which are related to aromatic C-H stretching vibration of 4-NPh. Besides, the intensity of the bands located at 1367 cm^−1^ and 1714 cm^−1^ increases due to the contribution of N-O stretching vibration in this region. These results shows that some of adsorbed 4-NPh molecules remain intact during the reaction and are not degraded by the photocatalyst. No additional bands appeared in the FT-IR spectrum of composite after removal of methylene blue, indicating its approximately complete degradation.

#### Kinetic studies of adsorption/removal processes

Adsorption kinetics is usually studied to explore the factors affecting the rate of the adsorption process. Pseudo-first-order and pseudo-second-order kinetic models quantitatively express the adsorption kinetics. To understand the kinetics of pollutant adsorption by prepared nanocomposites, both pseudo-first-order and pseudo-second-order kinetic models were studied. The results shown in Fig. S4 and Table 4 confirmed that all samples obey the pseudo-second-order model. The pseudo-second-order kinetic model is presented by Eq. (2), in which k (g mg^−1^ min^−1^) is the rate constant, and q_t_ and q_e_ (mg g^−1^) show the adsorbed amount of adsorbate at time t (min) and at equilibrium, respectively.

2 $$\frac{t}{{q_{t} }} = ~\frac{1}{{kq_{e}^{2} }} + ~\frac{1}{{q_{e} }}~t$$

q_t_ can be calculated using the Eq. (3):

3 $$q_{t} = ~\frac{{\left( {C_{0} - ~C_{t} } \right)~ \times V}}{W}$$

where, C_0_ and C_t_ (mg L^−1^) are the initial concentration of adsorbate and its concentration at time t, V (mL) is the volume of solution, and W (mg) shows the weight of adsorbent^54^.

According to the results, linear plots with the high R^2^ values were obtained for all samples using the pseudo-second-order kinetic model. The correlation coefficient is > 0.99 for most of the studied adsorbents and is equal to 1 for MB adsorption by Fe_3_O_4_20%-GQD/BZ. The pseudo-second-order kinetic model shows that chemisorption is the rate-limiting step which controls the adsorption process. In this model, the rate of adsorption sites’ occupation is proportional to the square of the unoccupied sites number.

**Table 4:** Shows that the adsorbed amount of pollutants at equilibrium (q_e_) for all two- and three-component nanocomposites is greater than that of bare borosilicate. Furthermore, three-component nanocomposites can adsorb more pollutants than two-component counterparts which can be attributed to their higher surface area. The maximum q_e_ belongs to Fe_3_O_4_20%-GQD/BZ nanocomposite, which possesses the largest BET surface area.

Sample/Parameters	Nitrate			MB			4-NPh		
	k_2_ (min^−1^)	q_e_ (mg g^−1^)	*R* ^2^	k_2_ (min^−1^)	q_e_ (mg g^−1^)	*R* ^2^	k_2_ (min^−1^)	q_e_ (mg g^−1^)	*R* ^2^
BZ	0.050	4.02	0.9952	0.018	5.29	0.9963	0.050	8.45	0.9882
GQD/BZ	0.075	4.10	0.9942	0.022	9.46	0.9968	0.015	16.31	0.9996
Fe_3_O_4_20%/BZ	0.035	4.72	0.9927	0.005	6.81	0.9675	0.004	13.33	0.9933
Fe_3_O_4_50%/BZ	0.015	5.65	0.9906	0.002	7.41	0.9738	0.006	14.35	0.9964
Fe_3_O_4_20%-GQD/BZ	0.010	7.22	0.9938	0.057	9.92	1	0.008	18.87	0.9994
Fe_3_O_4_50%-GQD/BZ	0.018	6.44	0.9948	0.047	9.91	0.9999	0.009	18.21	0.9996

Table 4. The key parameters of pseudo-second-order kinetic model for adsorption of pollutants by prepared samples.

#### Removal of BOD and COD from industrial wastewaters

In continuing our previous work^18^the performance of synthesized materials was evaluated in reducing BOD and COD values of industrial wastewaters. The wastewater of a dairy production unit was used for studying the BOD removal whereas a paint factory’s wastewater was chosen to investigate the reduction of COD. The wastewater of paint factory contains suspended solids and high COD levels. As shown in Table 5, the nanocomposites are capable of reducing both COD and BOD, however, the removal percent of COD is lower than that of BOD. On the other hand, the removal efficiencies are not significantly affected by the content of Fe_3_O_4_ nanoparticles in magnetic nanocomposites. Similar to the results obtained for removal of nitrate and organic pollutants in this work, the three-component nanocomposites showed the highest removal efficiencies. This can be attributed to their high surface area and higher number of adsorption sites. The removal efficiencies of BOD and COD by these nanocomposites are about 100% and 60%, respectively. Compared to the previously reported results, the removal efficiency of COD is relatively low which can be attributed to its high initial concentration (5000 g L^−1^). Deniz İzlen Çifçi^55^ reported a COD removal of 84.6% from textile wastewater using a Co-Fe co-doped cigarette filter based carbon (CoFe-CFC**)**. The adsorbent dosage and contact time were respectively 3 g L^−1^ and 120 min. In other work, UV/S_2_O_8_^2−^/Fe-Mn-textile waste system was used for treatment of textile wastewater containing 369 mg L^−1^ COD. A removal efficiency of 87% was attained after 120 min, applying 7.5 g L^−1^ adsorbent and 2 g L^−1^ S_2_O_8_^2^ under a 11 W UV light source^56^.

Daily monitoring of BOD values revealed that, unlike borosilicate zeolite, the active sites of synthesized nanocomposites are occupied by pollutants in the first hours of contact and no desorption is observed after several days.

**Table 5:** Nanoadsorbents performance in reducing BOD and COD from industrial wastewater.

Sample	Pollution index	Adsorbent amount(g)	Initial values (mg.L^−1^)	Final values (mg.L^−1^)	Removal%
BZ	BOD	0.1	98	29	70.41%
BZ	COD	0.3	5000	3160	36.8%
GQD/BZ	BOD	0.1	98	12	87.76%
GQD/BZ	COD	0.3	5000	2940	41.2%
Fe_3_O_4_20%-BZ	BOD	0.1	98	21	78.58%
Fe_3_O_4_20%-BZ	COD	0.3	5000	2640	47.2%
Fe_3_O_4_50%-BZ	BOD	0.1	98	16	83.68%
Fe_3_O_4_50%-BZ	COD	0.3	5000	2440	51.2%
Fe_3_O_4_20%-GQD/BZ	BOD	0.1	98	0	100%
Fe_3_O_4_20%-GQD/BZ	COD	0.3	5000	2000	60%
Fe_3_O_4_50%-GQD/BZ	BOD	0.1	98	0	100%
Fe_3_O_4_50%-GQD/BZ	COD	0.3	5000	2020	59.6%

• Experimental conditions for COD removal: pH: 6, Contact time: 2 h, Temperature: 25 °C.

#### Comparative assessment of adsorption/removal tests

The efficiency of optimal nanocomposite for the removal of nitrate, MB, 4-NPh, BOD, and COD from water and wastewater was compared with the previously reported adsorbents in Table 6. As seen, the synthesized nanocomposite exhibits versatile efficacy in the removal of diverse aqueous contaminants, with performance metrics comparable to existing benchmarks reported in the literature^24,57,58^.

**Table 6:** Comparison of adsorption capacity and other experimental conditions of several different adsorbent for nitrate, MB, 4-NPh, BOD, and COD removal.

Adsorbent	Adsorbate	Experimental conditions	Amount adsorbed	Reference
Chitosan-graft-poly(N-hydroxyethyl)acrylamide/Polyaniline/magnetite nanocomposite	Nitrate	pH: 3.0 Concentration range: 100 mg L^−1^ Contact time: 1 h Temperature: 30 °C	55.24 (mg g^−1^)	^59^
Fe-modified chitosan–zeolite	Nitrate	pH: 3.0 Concentration range: 25 mg L^−1^ Contact time: 40 min Temperature: 25 °C	5.18 (mg g^−1^)	^60^
Clinoptilolite Zeolites Loaded with Iron Oxide and Copper Oxide	Nitrate	pH: 7.0 Concentration range: 10 mg L^−1^ Contact time: 80 min Temperature: 25 °C	95%	^57^
(GQDs/Fe_3_O_4_/MMT)	MB	pH: 7.0 Concentration range: 100 mg L^−1^ Contact time: 20 min Temperature: 25 °C	93%	^24^
graphene quantum dots/ZSM-5 type metalosilicate composites	MB	pH: - Concentration range: 20 mg L^−1^ Contact time: 30 min Temperature: 25 °C	96%	^28^
KOH-modified coconut shell–clay composite (KCCC)	4-NPh	pH: 7.0 Concentration range: 300 mg L^−1^ Contact time: 8 h Temperature: 25 °C	476.9 (mg g^−1^)	^61^
Ce and Fe in the dealuminated Y zeolite (DAZY)	4-NPh	pH: 4.0 Concentration range: 5_*_10^−4^ M Contact time: 90 min Temperature: 25 °C	91%	^58^
Novel Zeolite 5Å-Co-Fe	BOD	pH:6 Concentration range: 122 mg L^−1^ Contact time: 15 min Temperature: 25 °C	55%	^62^
Granular Activated Carbon and Zeolite	COD	pH: 8 Concentration range: 1039.5 mg L^−1^ Contact time: 150 min Temperature: 25 °C	63.11%	^63^
Fe_3_O_4_20%-GQD/BZ nanocomposite	Nitrate	pH: 4.0 Concentration range: 30 mg L^−1^ Contact time: 90 min Temperature: 25 °C	85.51%	Present study
Fe_3_O_4_20%-GQD/BZ nanocomposite	MB	pH: - Concentration range: 20 mg L^−1^ Contact time: 150 min Temperature: 25 °C	98.28%	Present study
Fe_3_O_4_20%-GQD/BZ nanocomposite	4-NPh	pH: - Concentration range: 20 mg L^−1^ Contact time: 150 min Temperature: 25 °C	91.03%	Present study
Fe_3_O_4_20%-GQD/BZ nanocomposite	BOD	pH: 6 Concentration range: 98 mg L^−1^ Contact time: 2 h Temperature: 25 °C	100%	Present study
Fe_3_O_4_20%-GQD/BZ nanocomposite	COD	pH: 6 Concentration range: 5000 mg L^−1^ Contact time: 2 h Temperature: 25 °C	60%	Present study

#### Recyclability and reusability studies

The recyclability of Fe_3_O_4_50%-GQD/BZ composite was evaluated through successive adsorption/desorption cycles. For this purpose, the nanocomposite was subjected to nitrate adsorption experiment up to 3 cycles. After each experiment, the composite was recovered and washed with deionized water. To ensure the complete removal of adsorbed nitrate, the composite was stirred in deionized water for 10 min at room temperature. Then the composite was washed, dried and reused in the next cycle. The slight decrease (Fig. S5) in removal efficiencies after each cycle confirms the recyclability of prepared magnetic nanocomposite. This observation indicates that the nanocomposite can be easily recovered and reused in nitrate adsorption for several times. The high reusability of the adsorbent significantly reduces the overall cost of the adsorption process, making it a suitable candidate for industrial applications^64^. Considering the importance of nitrate-containing zeolites in agriculture, the nanocomposite can be used as a substitute for nitrate fertilizers after adsorption of nitrate.

## Conclusion

This work suggests a facile route for preparation of two-component and three-component nanocomposites with remarkable magnetic properties and high adsorption efficiency. MFI-type borosilicate zeolite and GQDs/borosilicate nanocomposite were used as supports for ultrasound assisted co-precipitation of Fe_3_O_4_ nanoparticles with 20 and 50 wt%. The obtained nanocomposites were tested for the removal of pollutants from water and wastewater and showed high adsorption capacities for removal of nitrate, 4-nitrophenol (4-NPh), methylene blue (MB), BOD, and COD. The best results were achieved using three-component nanocomposites (Fe_3_O_4_20%-GQD/BZ and Fe_3_O_4_50%-GQD/BZ) with surface areas of 464 and 338 m^2^g^−1^. FTIR spectra examination before and after pollutant adsorption by these nanocomposites revealed the appearance of peaks related to N-O stretching vibrations after nitrate adsorption and new peaks assignable to aromatic C-H stretching vibration after 4-nitrophenol adsorption. No additional peaks observed in the FTIR spectrum owing to methylene blue adsorption, indicating the photocatalytic degradation of dye. Kinetic studies showed that the adsorption of all three pollutants could be better described using the pseudo-second-order model. The high R-square value (R^2^ = 1) obtained for kinetic behavior of methylene blue adsorption indicates that chemisorption is the rate-limiting step.

The synthesized magnetic nanocomposites may be readily extracted from solution with a magnet and are effective candidates for the elimination of pollutants from water and wastewater.

## Electronic supplementary material

Below is the link to the electronic supplementary material.

Supplementary Material 1

## Data Availability

All data generated or analyzed during this study are included in this manuscript.
