# Supplementary material for: Synthesis of magnetic borosilicate zeolite/graphene quantum dots nanocomposites for removal of nitrate and organic pollutants from water
Source: Sci Rep. 2025 Jul 1;15:20726. doi: 10.1038/s41598-025-07746-4 (PMC12214790; doi:10.1038/s41598-025-07746-4)
Supplement: Supplementary file 1 — Supplementary Material 1 [file 41598_2025_7746_MOESM1_ESM.docx]

Supplementary Information

For

**Synthesis of magnetic borosilicate zeolite/graphene quantum dots nanocomposites for removal of nitrate and organic pollutants from water**

Robab Shahi^1^, Maasoumeh Khatamian^1 *^

^1^Department of Inorganic Chemistry, Faculty of Chemistry, University of Tabriz, Tabriz, Iran

^*^Corresponding author. Tel.: +98 413 3393129; Fax: +98 413 3340191

E-mail address: [Khatamian@tabrizu.ac.ir](mailto:Khatamian@tabrizu.ac.ir), [mkhatamian@yahoo.com](mailto:mkhatamian@yahoo.com) (Maasoumeh Khatamian).

**(a)**

**
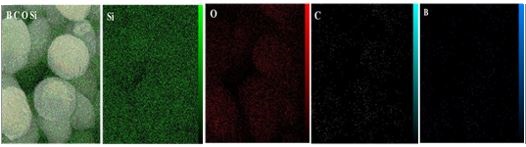
**

**(b)**


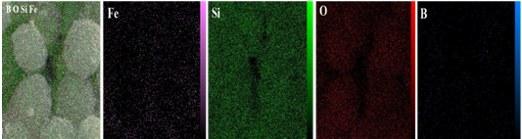


**
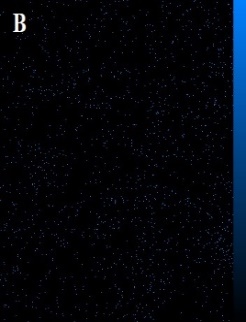

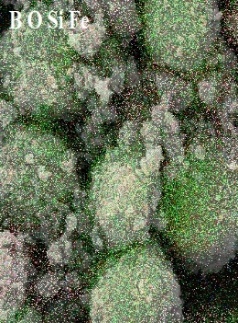
(c)
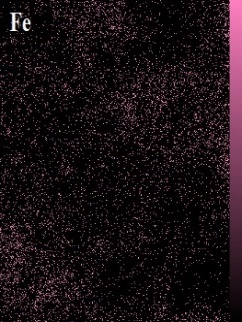

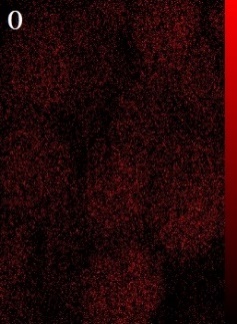

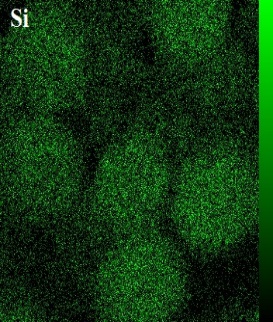
**

**(d)**


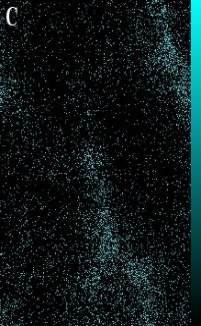

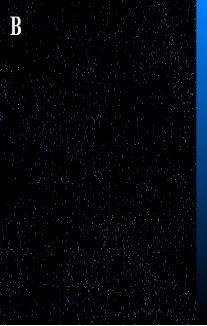

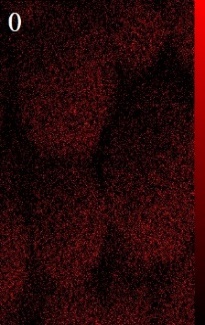

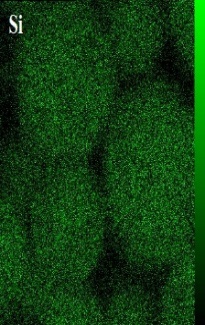

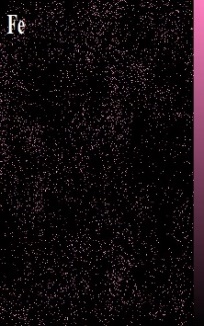

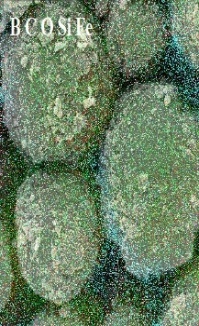


**(e)**

*
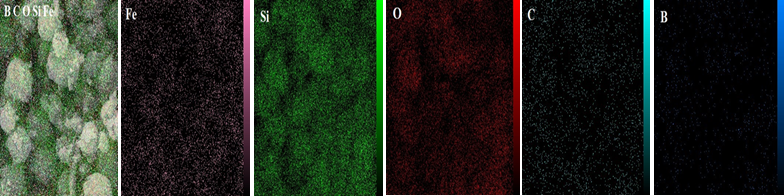
*

**Fig. S1.** Element distribution mappings of B, C, O, Si and Fe in GQD/BZ (**a**), Fe_3_O_4_20%-BZ (**b**), Fe_3_O_4_50%-BZ (**c**), Fe_3_O_4_20%-GQD/BZ (**d**), and Fe_3_O_4_50%-GQD/BZ (**e**) nanocomposites.


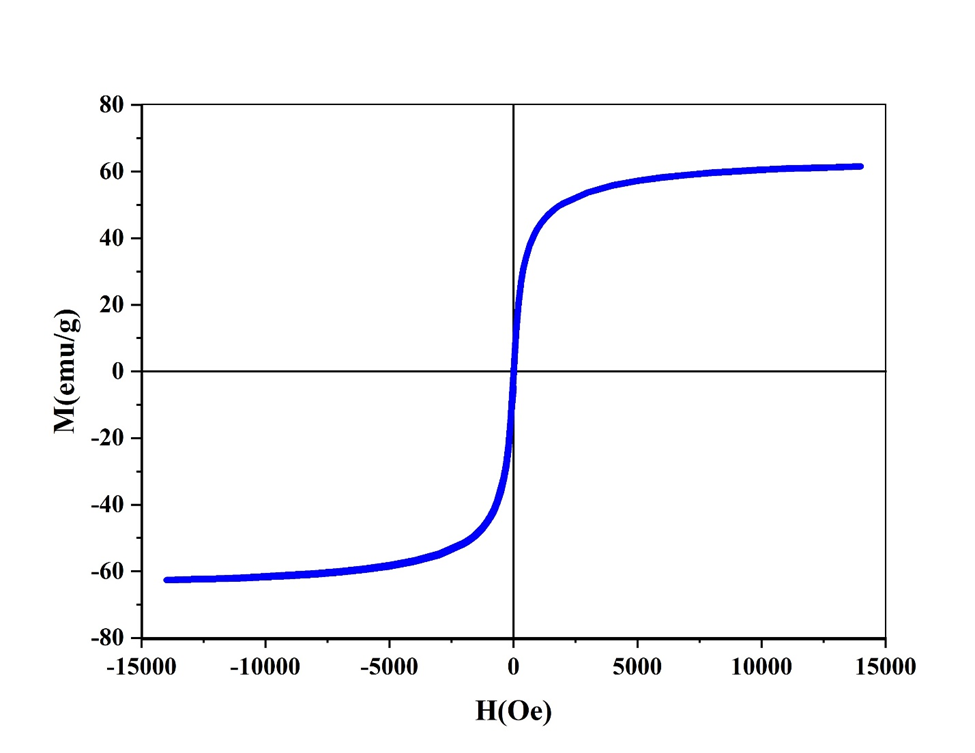


**Fig. S2.** M-H curve of Fe_3_O_4_ nanoparticles.


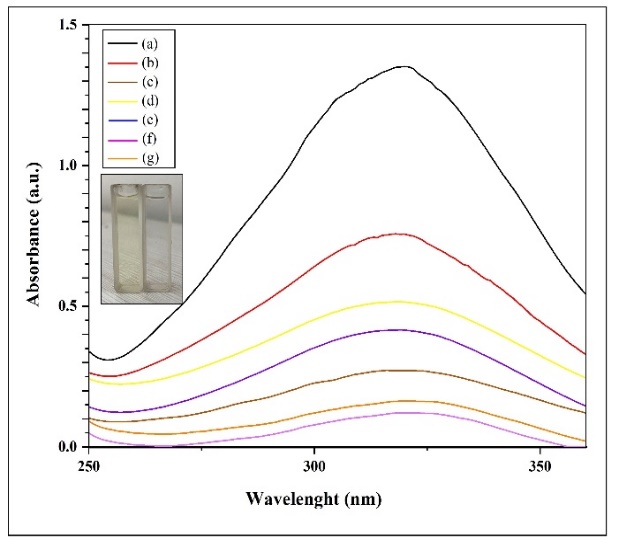

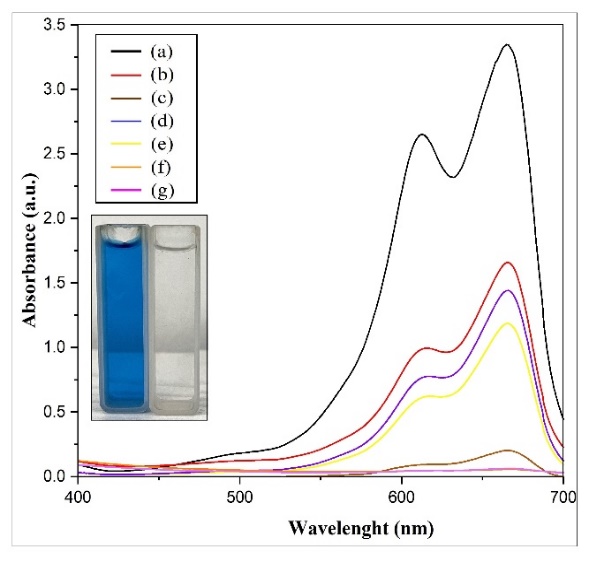


**Fig. S3.** UV-vis absorption spectra of MB (left) and 4-NPh (right) stock solutions (20 ppm) (**a**) and corresponding solutions after photocatalytic removal by borosilicate zeolite (**b**), GQD/BZ (**c**), Fe_3_O_4_20%-BZ (**d**), Fe_3_O_4_50%-BZ (**e**), Fe_3_O_4_20%-GQD/BZ (**f**), and Fe_3_O_4_50%-GQD/BZ (**g**).


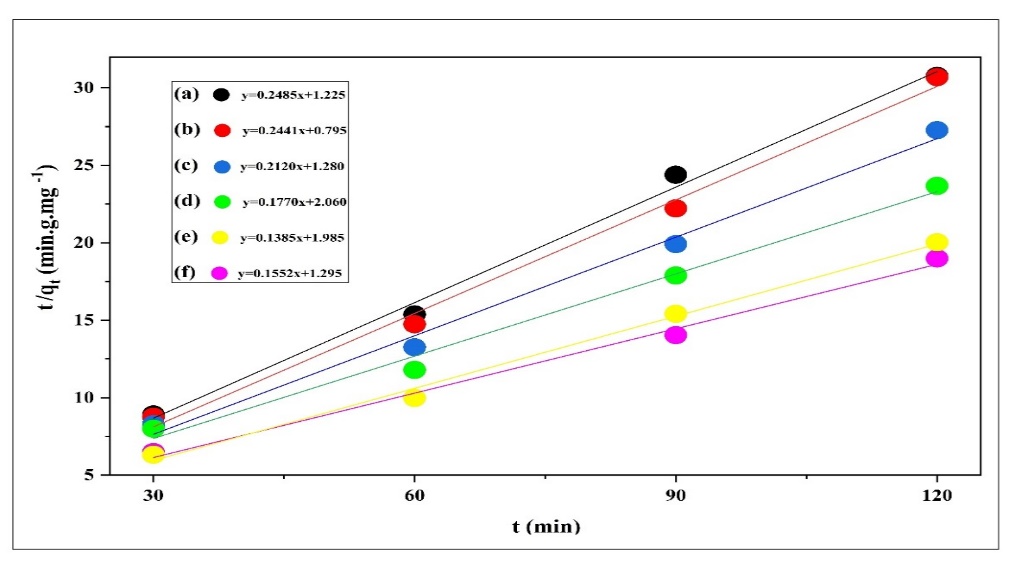


(A)


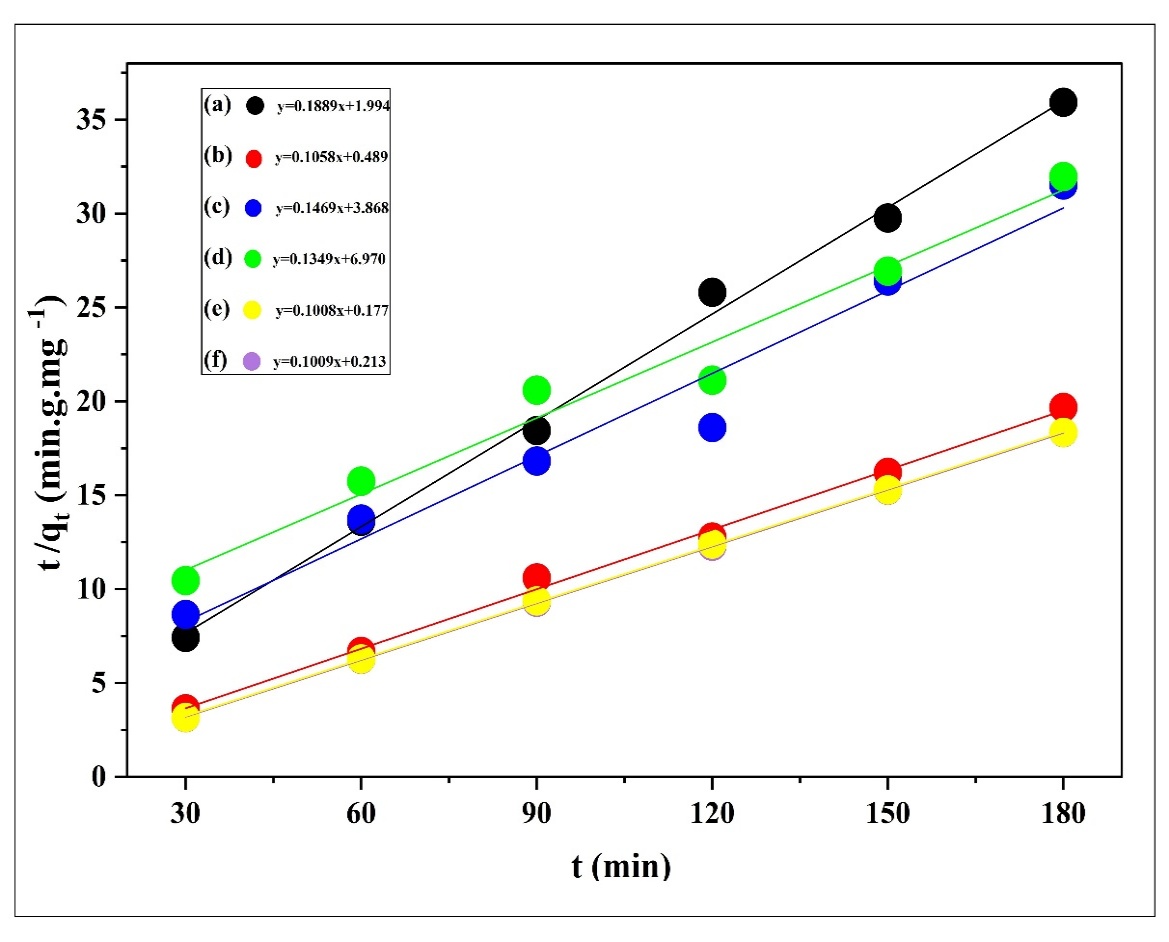


(B)


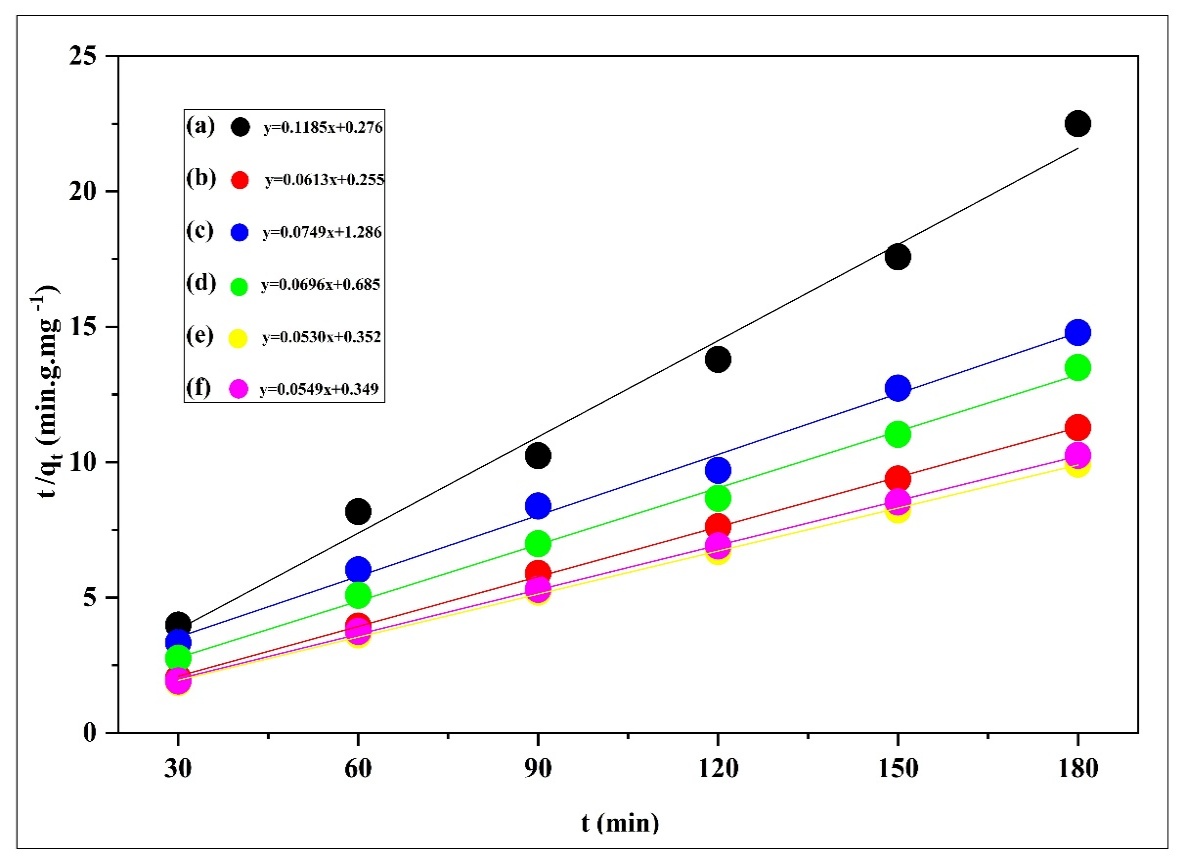


(C)

**Fig. S4.** Plots of the pseudo-second-order kinetic model for adsorption of nitrate (**A**), methylene blue (**B**), and 4-nitrophenol (**C**) by borosilicate (**a**), GQD/BZ (**b**), Fe_3_O_4_20%-BZ (**c**), Fe_3_O_4_50%-BZ (**d**), Fe_3_O_4_20%-GQD/BZ (**e**), and Fe_3_O_4_50%-GQD/BZ (**f**).


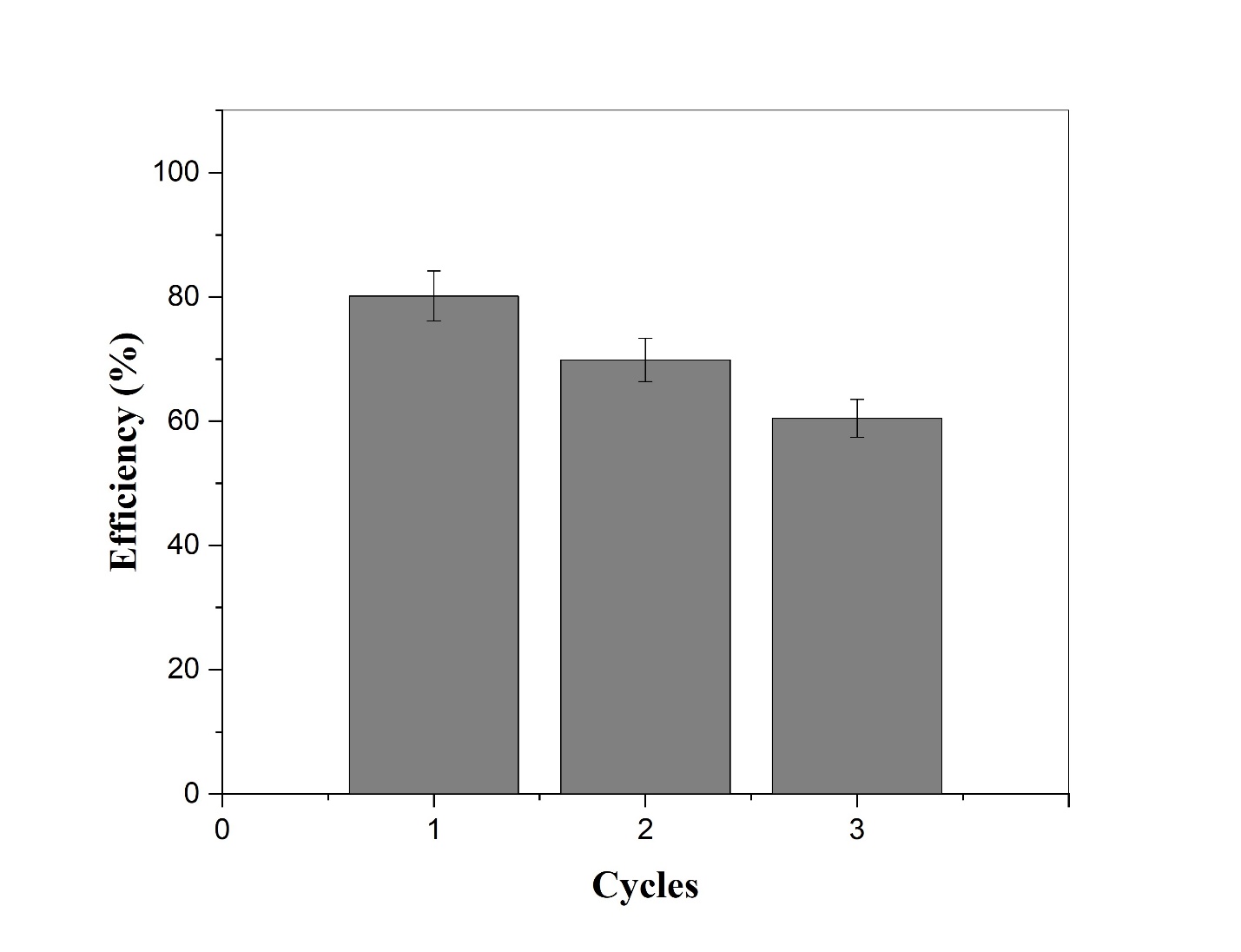


**Fig. S5.** Reusability of Fe_3_O_4_50%-GQD/BZ nanocomposite in nitrate adsorption.
